# Photoswitchable Calixarene Activators for Controlled Peptide Transport across Lipid Membranes

**DOI:** 10.1021/jacs.3c01829

**Published:** 2023-06-08

**Authors:** Joana N. Martins, Beatriz Raimundo, Alicia Rioboo, Yeray Folgar-Cameán, Javier Montenegro, Nuno Basílio

**Affiliations:** † Laboratório Associado para a Química Verde (LAQV), Rede de Química e Tecnologia (REQUIMTE), Departamento de Química, Faculdade de Ciências e Tecnologia, Universidade NOVA de Lisboa, 2829-516 Caparica, Portugal; ‡ Centro Singular de Investigación en Química Biolóxica e Materiais Moleculares (CIQUS), Departamento de Química Orgánica, Universidade de Santiago de Compostela, 15782 Santiago de Compostela, Spain

## Abstract

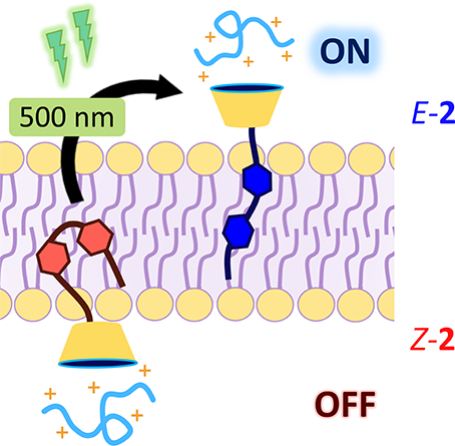

Supramolecular synthetic transporters are crucial to understand and activate the passage across lipid membranes of hydrophilic effector molecules. Herein, we introduce photoswitchable calixarenes for the light-controlled transport activation of cationic peptide cargos across model lipid bilayers and inside living cells. Our approach was based on rationally designed *p*-sulfonatocalix[4]arene receptors equipped with a hydrophobic azobenzene arm, which recognize cationic peptide sequences at the nM range. Activation of membrane peptide transport is confirmed, in synthetic vesicles and living cells, for calixarene activators featuring the azobenzene arm in the *E* configuration. Therefore, this method allows the modulation of the transmembrane transport of peptide cargos upon *Z*–*E* photoisomerization of functionalized calixarenes using 500 nm visible light. These results showcase the potential of photoswitchable counterion activators for the light-triggered delivery of hydrophilic biomolecules and pave the way for potential applications in remotely controlled membrane transport and photopharmacology applications of hydrophilic functional biomolecules.

## Introduction

Selective transport of ions, metabolites, and large biomolecules across lipid membranes is a fundamental process for the maintenance of cellular function and homeostasis in living organisms.^1^ While small, moderately polar molecules can spontaneously cross cell membranes by passive diffusion, the translocation of hydrophilic substances with low permeability, including biologically relevant ions and large biomolecules, is generally accomplished through a variety of transport mechanisms involving stimulus-responsive membrane proteins. These biomolecular machines and active transporters are frequently claimed as a source of inspiration for the development of artificial supramolecular channels and carriers.^2−7^ Synthetic transporters, usually designed as simplified prototypes of their biological counterparts, are useful surrogates to investigate membrane transport mechanisms and for use as active pharmaceutical ingredients or drug-delivery systems.^2,4−12^

Stimulus-responsive artificial transporters displaying photomodulated activity are particularly appealing due to the advantages of using light as a stimulus, which include remote application, a high degree of spatiotemporal precision, and, in most cases, no production of chemical waste.^2,13,14^ Their utility for biological and pharmaceutical applications has led to the successful development of a considerable number of photoresponsive artificial channels and pores operating through different mechanisms.^2^ However, independently of their functional mechanism, most photoresponsive transporting systems are designed to target small ions, while those directed to larger biomolecules remain elusive.^15−24^

Among the different systems developed to transport large hydrophilic cargos across membranes, amphiphilic counterion activators have proven to provide efficient synthetic carriers using relatively simple and synthetically accessible molecules.^8,25^ Counterion activation phenomena result from the binding of polyionic species with oppositely charged molecules, forming charge-neutralized complexes with higher membrane permeability.^8^ Fundamental studies on counterion-activated membrane transport have been frequently carried out using cationic peptides as cargo molecules.^26−31^ These investigations have made important contributions to elucidate the intriguing high membrane permeability of polycationic peptides, showing that their dynamic association with anionic molecules present in cell membranes (e.g., anionic lipids or glycosaminoglycans) plays an important role in their transport mechanism.^8,32^ Systematic screenings have identified pyrene carboxylate amphiphiles as prime activators for the transport of poly- and oligoarginines across phosphatidylcholine lipid bilayers.^26,29^ Amphiphilic sulfonatocalixarenes have also been shown to be highly efficient counterion activators for the membrane transport of cationic peptides.^33−35^

**Scheme 1:**
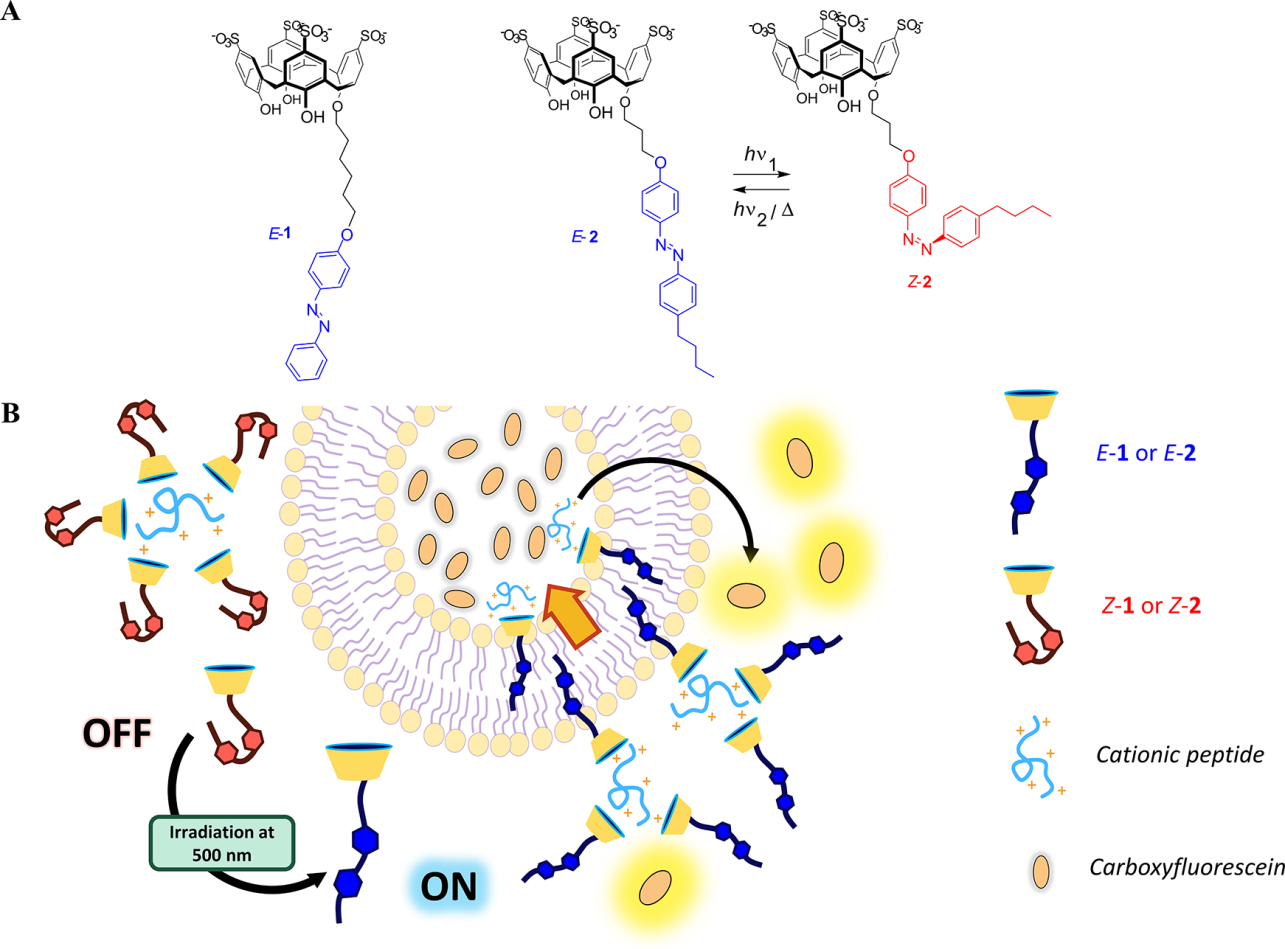
(A) Molecular Structure of the Photoresponsive Counterion Activators Investigated in This Work and (B) Schematic Representation of the Phototriggered Transport of Cationic Peptides across Phospholipidic Membranes The relevant *E*–*Z* photoisomerization is illustrated with activator **2**. Both compounds were synthesized as lithium salts.

The excellent translocation activity of these anionic macrocyclic receptors was correlated with their binding affinity toward positively charged peptides and their membrane partitioning properties, with moderately amphiphilic receptors affording better activators.^33^ Previous comprehensive structure–activity studies have reported that subtle variations on the electronic and structural nature of activators can lead to dramatic changes in their transport activity.^27,29,30,36^ Consequently, the dynamic control of the structure/polarity of counterion activators offers a promising conceptual strategy to develop stimulus-responsive membrane carriers. We hypothesized that azobenzene photoswitches, which are known to experience significant conformational and polarity changes (Δμ ≈ 3*D*) upon *E*–*Z* photoisomerization,^37,38^ would provide a rational design motif for the potential construction of new light-responsive membrane transporters. In this work, we report the first synthesis of such photoswitchable counterion activators for the membrane translocation of hydrophilic peptide cargos. Our design relied on sulfonatocalix[4]arene-azobenzene conjugates (Scheme 1A), as anionic receptors with photomodulated amphiphilicity, which enables the light-triggered transport activation of cationic peptides across artificial lipid bilayers and inside living cells (Scheme 1B).

## Results and Discussion

The synthesis of the photoresponsive counterion activators **1** and **2** was accomplished by direct monoalkylation of the sulfonatocalix[4]arene (SC4) macrocycle (the more organo-soluble lithium salt)^39^ with the respective azobenzene precursor in DMSO (see the Supporting Information). The ^1^H NMR results of both compounds in CD_3_OD (Figures S1 and S6) show two pairs of doublets for the four methylene bridges indicating that, in this solvent, **1** and **2** adopt the cone conformation.^40^ In D_2_O, the ^1^H NMR spectra of **1** and **2** (Figures S2 and S7) are substantially different from those observed in CD_3_OD, showing broader resonances, which can be tentatively assigned to intermediate conformational and/or aggregation exchange dynamics on the NMR chemical shift timescale.^41^

**Figure 1:**
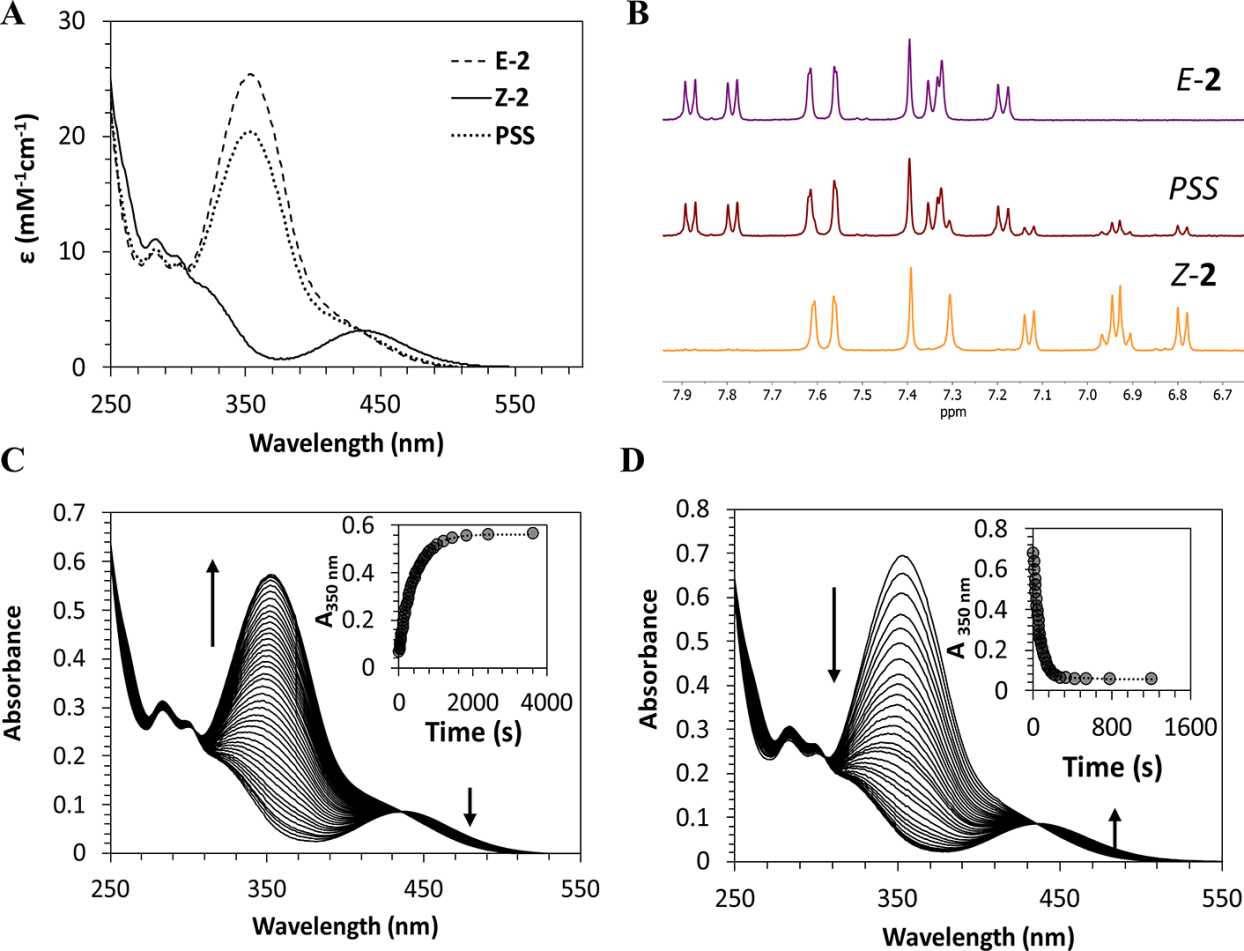
(A) Absorbance spectra of both *E* and *Z* isomers of **2** and the photostationary state (PSS; 80:20 *E*:*Z*) obtained after irradiation at 500 nm. (B) Partial ^1^H NMR (in CD_3_OD) spectra of the aromatic region of **2** at the dark-adapted state (*E* isomer), PSS upon irradiation at 500 nm (*E*:*Z* 80:20) and at 366 nm (*Z* isomer). (C) *Z* → *E* and (D) *E* → *Z* photoisomerization of **2** followed by UV–vis spectroscopy upon irradiation at 500 and 366 nm.

The photochromic properties of **1** and **2** were investigated in diluted aqueous solution by UV–vis absorption spectroscopy and in CD_3_OD by ^1^H NMR. Photochemical irradiation using a UV-light wavelength (λ_irr_) of 366 nm led to quantitative conversion of the *E* isomers into the respective *Z*-forms (Φ_**1***E → Z*_ = 0.4 and Φ_**2***E* → *Z*_ = 0.4) (see Figure 1 and Figure S23).

On the other hand, irradiation of the *Z*-forms at λ_irr_ = 500 nm originates a photostationary state (PSS) composed of 70 and 80% of *E*-**1** (**Φ**_**1***Z → E*_ = 0.6) and *E*-**2** (**Φ**_**2***Z → E*_ = 0.5), respectively (Figure 1 and Figure S23). As generally observed for other azobenzene photoswitches,^42^ the *E* isomers can be quantitatively recovered in the dark through the thermal *Z* → *E* isomerization, with **1** and **2** presenting activation energies of 215 and 93 kJ/mol, respectively (Figures S24 and S25). Despite the substantial difference in activation energies, both *Z*-**1** and *Z*-**2** are metastable species, presenting minimal thermal interconversion at room temperature during the time frame of our experiments, allowing for their use in binding studies and counterion activation assays without interferences from the thermal *Z* → *E* isomerization.

**Figure 2:**
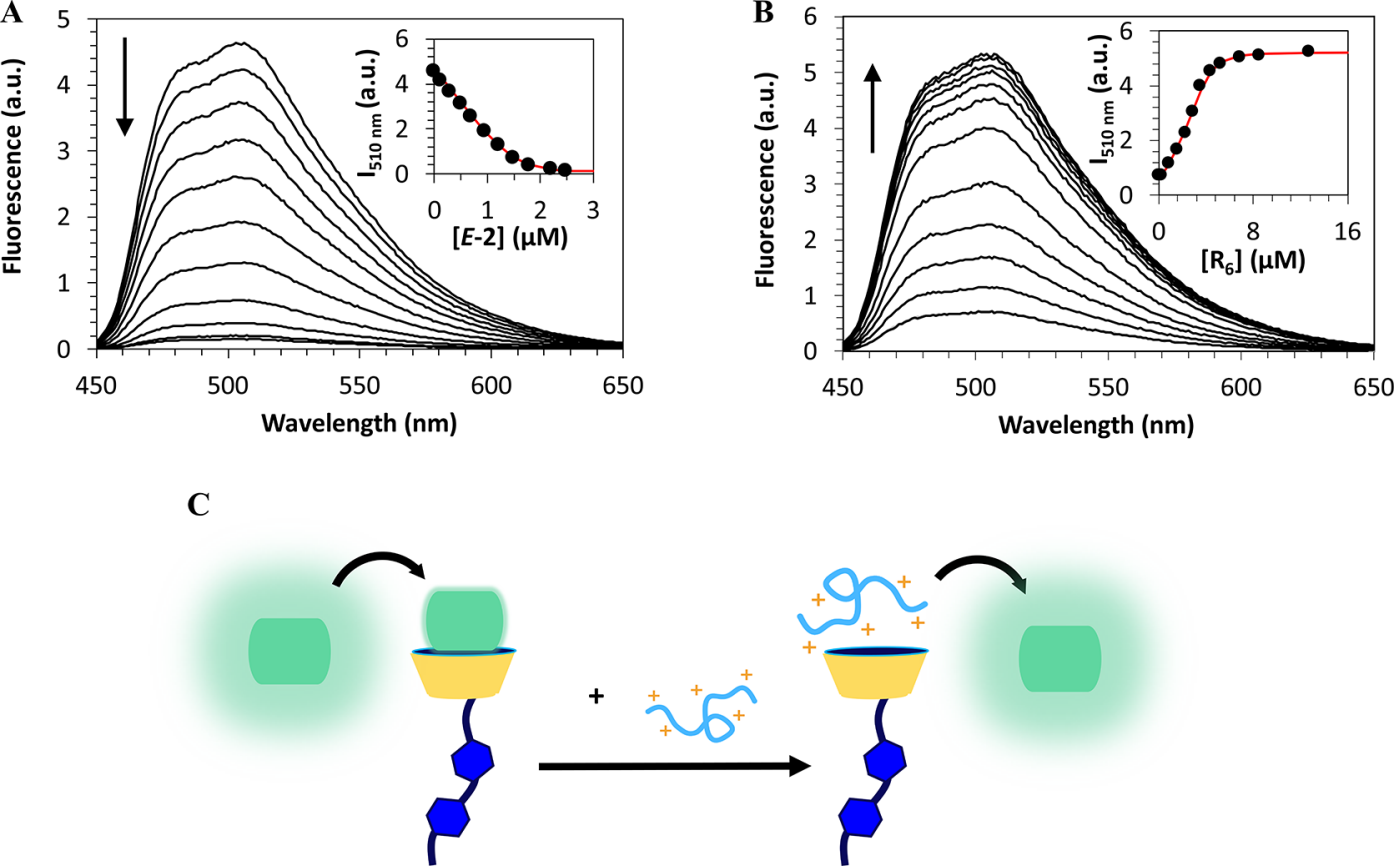
(A) Fluorescence titration of the lucigenin dye (LCG, 1.5 μM) with increasing concentrations of *E*-**2** in water (5 mM phosphate buffer, pH 7.3). (B) Indicator displacement titration of hexaarginine (R_6_) using *E*-**2**:LCG as the reporter pair (5 mM phosphate buffer, pH 7.3). LCG and *E*-**2** concentrations were fixed at 4 and 4.3 μM, respectively. (C) Schematization of the indicator displacement assay illustrating the formation of the dye:*E*-**2** complex and subsequent removal of LCG by the addition of peptides to the mixture, leading to the observed regaining of emission in (B).

The binding affinity of the anionic calixarene activators toward cationic peptides was investigated using a small library of highly hydrophilic oligoarginines, from 3 to 8 residues. These peptides are not able to spontaneously transverse zwitterionic artificial membranes in vesicle assays, which allows the accurate evaluation of the calixarenes **1** and **2** as counterion activators without interferences from the transport of the peptide alone.^43−45^ First, the formation of host–guest complexes between **1**/**2** and the selected peptides was verified by indicator displacement assays using lucigenin (LCG) as the fluorescent indicator.^46^ As previously reported for other sulfonatocalixarene derivatives,^46^ the formation of host–guest binding pairs between calixarenes **1**/**2** in the *E*- and *Z*-forms and LCG results in significant static fluorescence quenching (see Figure 2A and Figures S26 and S27). Quantitative analysis of the fluorescence titration data using a 1:1 host:guest binding model allows the determination of the respective binding constants (*K*) reported in Table 1. As can be observed, both **1**/**2** display high affinity for the LCG dye, on the 10^7^ M^–1^ range, which seems to be independent of the nature and conformation of the azobenzene arm. Furthermore, the observed binding constants are only slightly smaller than the one observed for the parent SC4 receptor (*K* = 8.1 × 10^7^ M^–1^, see Figure S28), suggesting that these monofunctionalized receptors retain their recognition properties. The host:LCG binding pairs were then employed in the above-mentioned indicator displacement assays to investigate the binding affinity of oligoarginines toward **1** and **2** (see Figure 2B and Figures S29–S33). The obtained results (see Table 1) show that both **1** and **2** display high affinity toward oligoarginines with binding constants that increase with the number of arginine residues (at least up to R_6_), reaching the nM range for larger, highly positively charged peptides.

**Table 1:** Apparent Affinity Constants (K/M^–1^) Calculated by Competitive Dye Displacement Assays Using Lucigenin (LCG) as a Fluorescence Probe for the SC4 Receptor and Counterion Activators **1** and **2**^b^

guest	SC4	*E*-**1**	*Z*-**1**	*E*-**2**	*Z*-**2**
LCG	(8.1 ± 0.9) × 10^7^	(2.5 ± 0.7) × 10^7^	(2.3 ± 0.4) × 10^7^	(2.6 ± 0.7) × 10^7^	(3.4 ± 0.8) × 10^7^
R_3_	(5.5 ± 0.3) × 10^6^	(2.6 ± 0.8) × 10^6^	(2.4 ± 0.6) × 10^6^	(4.1 ± 0.2) × 10^6^	(3.6 ± 0.3) × 10^6^
R_4_	(2.7 ± 0.4) × 10^8^	(3.9 ± 0.3) × 10^7^	(3.2 ± 0.7) × 10^7^	(1.2 ± 0.6) × 10^8^	(7.8 ± 1.3) × 10^7^
R_6_	>1 × 10^9^^a^	>5 × 10^8^^a^		>5 × 10^8^^a^	
R_8_	>1 × 10^9^^a^	>5 × 10^8^^a^		>5 × 10^8^^a^	

a Only the lower limit of *K* can be estimated because the titration curves display a sharp leveling-off at 1 equiv of the peptide, which is characteristic of very high binding affinity, precluding the accurate determination of *K* under the experimental conditions.

b All experiments were carried out in 5 mM phosphate buffer at pH 7.3. Standard deviations were obtained from triplicate experiments (see the SI).

Having shown that **1** and **2** bind the target peptides with high affinities and that the binding does not show significant dependence on the *E*/*Z* conformation (see Table 1, R_3_ and R_4_), the next step was to evaluate their effectiveness as photoresponsive counterion activators, using egg phosphatidylcholine large unilamellar vesicles (EYPC-LUV) as model zwitterionic membranes. The slightly negative ζ potential of EYPC-LUV shifts to more negative values with the addition of increasing concentrations of the anionic calixarene activators **1** and **2**, suggesting that these amphiphilic species are embedded in the lipid membrane (Figure S34). Dynamic light scattering (DLS) control experiments also confirmed liposome integrity under the experimental conditions employed in the different membrane transport assays, which ruled out potential membrane disruption interferences (Figure S34). Established dye efflux assays were performed using the carboxyfluorescein (CF) dye encapsulated at self-quenching concentrations in egg phosphatidylcholine large unilamellar vesicles (EYPC-LUV⊃CF).^26,27,29,45^ In these experiments, the fractional release of trapped CF (or fractional transport activity, *Y*), triggered by the addition of counterion activators and peptides, is monitored by time-resolved fluorescence spectroscopy and calibrated by the addition of Triton X-100 (Figure S35).

**Figure 3:**
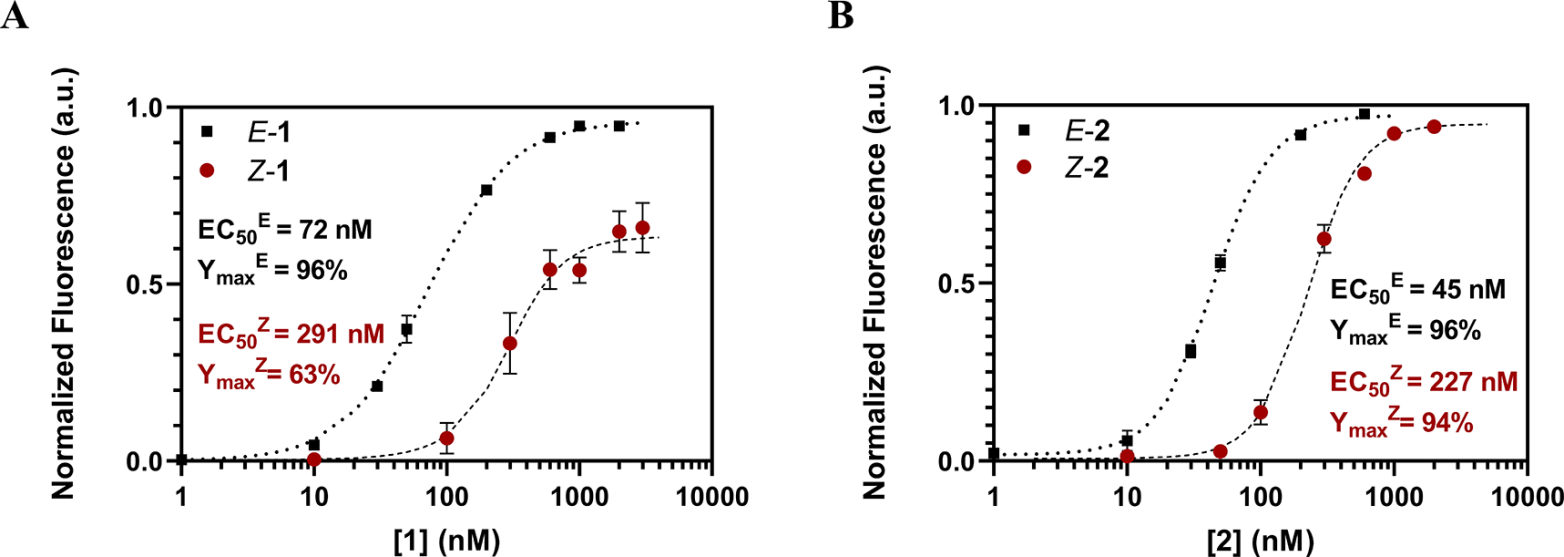
Concentration-dependent counterion transport activity obtained from CF efflux assays for (A) *E*-**1**/*Z*-**1** and (B) *E*-**2**/*Z*-**2** at a constant R_6_ concentration (4 μM). The experimental data were fitted to the Hill equation (dotted and dashed lines).

The studies were performed with both **1** and **2** in the *E* and *Z* configurations in the presence of fixed concentrations of liposomes and cationic peptides. It should also be noted that in the absence of peptides, the addition of counterion activators does not affect the intensity of the fluorescence signal, discarding the leakage of the vesicle contents by **1** or **2** alone (Figure S35). Likewise, the addition of peptides alone or nonbinding anionic peptides in the presence of calixarene activators also results in unmodified fluorescence signal, confirming that the formation of the calixarene–peptide complex is critical to activate the transport process. Transport activity was observed in the presence of peptides containing four, six, or eight arginine residues. Although complete sigmoidal dose–response curves could not be obtained for **1** and **2** in the presence of R_4_ (see Figure S36), the results show a higher efficiency of *E* isomers to translocate this peptide across the lipid membrane, supporting the hypothesis that these calixarene-based activators can be used in light-activated transport assays. In contrast to R_4_, R_6_ (Figure 3) and R_8_ (Figure S37) allow quantitative analysis of the resulting concentration-dependent transport activity plots by fitting these data to the Hill equation.^26,27,29,45^ This approach retrieves the *Y*_max_ (i.e., maximal CF release relative to liposome lysis after the addition of Triton X-100) and EC_50_ (i.e., the effective counterion activator concentration required to reach *Y*_max_/2) as the relevant parameters to measure the efficiency of the counterion activators. Because counterion activators with low EC_50_ do not necessarily display high *Y*_max_, Matile and co-workers proposed an equation, TE = *Y*_max_ × pEC_50_/*f*, to evaluate the transport efficiency (TE) as a function of *Y*_max_ and the negative logarithm of the EC_50_ (pEC_50_), with *f* = 20.6 being a scaling factor.^27^

The results summarized in Table 2 demonstrate that, in line with previous studies of anionic amphiphilic calixarenes,^33,35^ the monofunctionalized calixarenes **1** and **2** are highly efficient counterion activators. In all cases, the *E* isomers showed lower EC_50_ and generally higher *Y*_max_ values than their corresponding *Z* analogs. This indicates that, although both *E* and *Z* calixarenes counterions can activate the transport of cationic peptides, the *E* isomer shows an enhanced membrane transport activation (by a factor of up to 5-fold in EC_50_, Figure 3 and Table 2). Here, the counterion selectivity combined with the photoswitchable activation properties of **1** and **2** sets favorable conditions for the development of light-activated transporters for cationic peptides across phospholipid membranes.

**Table 2:** Counterion Activation Efficiency Parameters of Amphiphilic Sulfonatocalixarenes **1** and **2** in the *E* and *Z* Conformations for the R_6_ and R_8_ Transport across Egg Phosphatidylcholine Large Unilamellar Vesicles (EYPC-LUV⊃CF)^c^

peptide	activator	EC_50_^a^ (nM)	*Y*_max_^a^ (%)	TE^b^
R_6_	*E*-**1**	72 ± 5	96 ± 1	19.4 ± 0.2
	*Z*-**1**	291 ± 32	63 ± 5	10.8 ± 0.8
	*E*-**2**	45 ± 2	96 ± 1	20.2 ± 0.2
	*Z*-**2**	227 ± 17	94 ± 2	16.6 ± 0.3
R_8_	*E*-**1**	24 ± 2	91 ± 2	20.3 ± 0.4
	*Z*-**1**	99 ± 3	69 ± 2	13.3 ± 0.4
	*E*-**2**	11 ± 1	93 ± 2	22.2 ± 0.3
	*Z*-**2**	30 ± 4	94 ± 2	20.8 ± 0.4

a Obtained from the fitting of the Hill equation to the experimental dose-response data (e.g., data shown in Figure 3 and in the Supporting Information).

b Calculated from TE = *Y*_max_ × pEC_50_/*f*, with pEC_50_ = −log EC_50_ (in mM) and *f* = 20.6.

c All experiments were carried out in 5 mM phosphate buffer at pH 7.3, with a fixed concentration of peptides and EYPC-LUV⊃CF at 4 and 15 μM, respectively, for all experiments. Standard deviations were obtained from triplicate experiments (see the SI).

**Figure 4:**
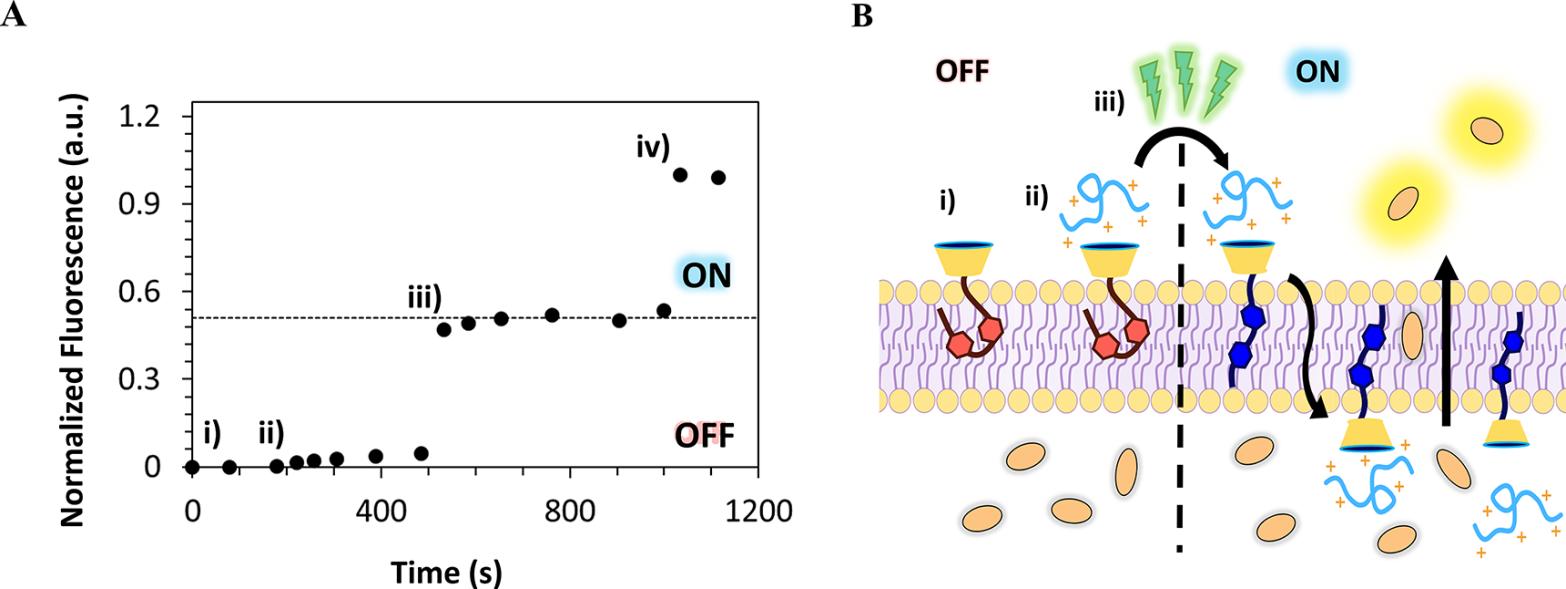
(A) Light-modulated dye efflux assays performed with 4 μM R_6_, 60 nM *Z*-**2**, and 15 μΜ EYPC-LUV⊃CF (50 mM CF inside the liposome) and (B) schematization of each phase in the assay. (i) First, the activator *Z*-**2** was added to a liposome solution (ii) followed by the addition of the peptide. (iii) The same sample was irradiated for 25 min at 500 nm, leading to a significant increase in CF release. (iv) Finally, in order to normalize the measurement for the maximum emission possible, the vesicles were lysed by addition of a small aliquot of Triton X-100.

CF efflux assays, similar to those described above, were also performed to demonstrate photoinduced peptide transport across phospholipid membranes using calixarene-based counterion activators. To this aim, *Z*-**1** and *Z*-**2** were added to EYPC-LUV⊃CF dispersions at fixed concentrations to study the CF release upon *in situ* light stimulation. As illustrated in Figure 4, the addition of the peptide in the presence of *Z*-**2** leads to a small increase in the CF fluorescence intensity, in agreement with the translocation of a residual fraction of R_6_ across the lipid bilayer. Irradiation with 500 nm light promotes the conversion of *Z*-**2** into *E*-**2**, a stronger counterion activator, leading to ca. 50% of extra dye released as a result of the light-triggered translocation of R_6_ into the liposome (Figure 4 iii). These results were further confirmed by analogous experiments carried out with the **2**-R_8_, **1**-R_6_, and **1**-R_8_ activator-peptide pairs (see Figures S44 and S45).^47^

**Figure 5:**
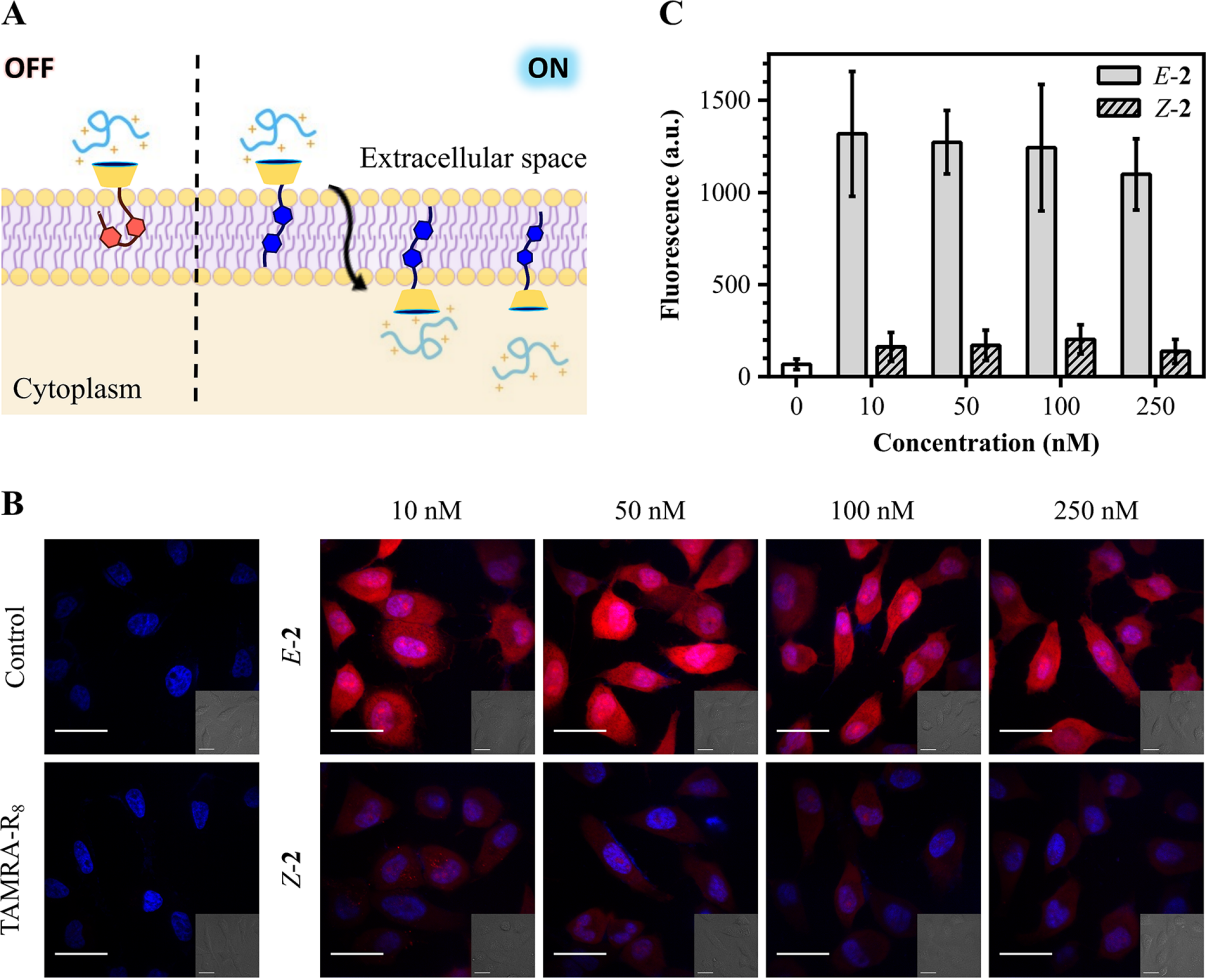
(A) Schematic representation of oligoarginine intracellular delivery by both isomers of **2**. (B) Confocal fluorescence microscopy images of the cellular uptake of 3 μM TAMRA-R_8_ (in red) facilitated by *E*-**2**/*Z*-**2**. Hoechst stained nuclei can be seen in blue and differential interference contrast (DIC) images are presented as insets.. Control experiments of TAMRA-R_8_ in the absence of a calixarene activator are also included. Scale bars, 50 μm. (C) Fluorescence intensity of HeLa cells incubated with 3 μM TAMRA-R_8_ in the presence of different *E*-**2**/*Z*-**2** concentrations, measured by flow cytometry.

Success in artificial vesicle assays encouraged us to conduct transport experiments of fluorescently labeled peptides in living cells (Figure 5). We first employed confocal fluorescence microscopy to evaluate counterion activator **2** for the intracellular transport of a carboxytetramethylrhodamine-labeled R_8_ peptide (TAMRA-R_8_) into the cytosol of HeLa cells. Cationic peptides usually remain trapped inside the endosomes when incubated with cells at low μM concentrations.^32,44^ As expected, control experiments at a fixed TAMRA-R_8_ low concentration (3 μM) and in the absence of calixarene activators confirmed an almost negligible cytosolic signal from the labeled peptide (Figure 5B and Figure S51). However, in agreement with vesicle assays, the *E* isomer of the counterion activator exhibited a significant increase of the intracellular delivery as compared to the less hydrophobic *Z* configuration (Figure 5B). Confocal micrographs of cells incubated with TAMRA-R_8_, in the presence of the *E* isomer of the calixarene activator **2**, revealed a strong diffuse fluorescence signal in the cytosol, nucleus, and nucleolus of the cells (Figure 5B). To validate and compare these results, flow cytometry quantification of peptide uptake was carried out in the presence of both *Z* and *E* isomers of the calixarene **2**. The cytometry experiments validated the *E* isomer selective transport and indicated a four-fold intracellular delivery enhancement (Figure 5C and Figure S52). Although the trend of activity of the two calixarene isomers was maintained in vesicles and cell assays, the observed variations on the EC_50_ values can be due to the different experimental conditions, membrane composition, and competing energy-dependent internalization pathways.^27,29,36,48^ Additionally, the MTT viability assay confirmed a low cellular toxicity for the counterion activators at the employed concentration regime (Figure S53). The IC_50_ cytotoxicity values, of 16.88 μM for *E*-**2** and of 19.16 μM for *Z*-**2** (after 24 h of incubation), appeared 2–3 orders of magnitude higher than the nM activator concentrations, which were required for an efficient peptide cytosolic delivery with the *trans* isomer of the calixarene counterion activator (*E*-**2**). The anionic nature of the calixarene activator should prevent nondesired interactions with other components of the cell (glycosaminoglycans, anionic lipids, ATP, etc.). While parallel transport events of other charged biomolecules cannot be completely ruled out, those processes do not cause any limitation to cellular viability and efficient cargo transport (Figure S53).

## Conclusions

In conclusion, we here introduce the first photoswitchable amphiphilic counterion activators for the transport of cationic peptides across lipid membranes and inside cells. We have synthetized calixarene-based receptors monofunctionalized with an azobenzene unit that can be applied as photoresponsive counterions to gain control over the transport of cationic peptides across artificial lipid membranes and into the cytosol of living cells. Our design combines a high-affinity oligoarginine binding unit, based on a sulfonatocalixarene macrocyclic host decorated with azobenzene pendants, whose lipophilicity can be remotely controlled by light-induced interconversion between the more hydrophobic, extended *E* and the more polar, bent *Z* configuration. The transport efficiency of these light-sensitive counterion activators was significantly enhanced for the *E* azobenzene isomers, which allows their application as photomodulated carriers for hydrophilic cargos. Based on the robust performance and response to light stimulation of these molecular photoswitches, we envisage possibilities in conceptually new stimulus-responsive transport systems for biological, pharmaceutical, and analytical applications.

## Supporting Information Available

Experimental details, general methods, synthetic procedures, and characterization of synthesized compounds; detailed photochemical characterization of light-responsive compounds, additional binding studies, dye efflux, and biological assays (PDF)
