# Supplementary material for: Photoswitchable Calixarene Activators for Controlled Peptide Transport across Lipid Membranes
Source: J Am Chem Soc. 2023 Jun 8;145(24):13126–33. doi: 10.1021/jacs.3c01829 (PMC10288505; doi:10.1021/jacs.3c01829)
Supplement: Supplementary file 1 — ja3c01829_si_001.pdf [file ja3c01829_si_001.pdf]

# Photoswitchable Calixarene Activators for Controlled Peptide Transport Across Lipid Membranes.

Joana N. Martins,‡ Beatriz Raimundo,‡ Alicia Rioboo,† Yeray Folgar-Cameán, † Javier Montenegro,* † Nuno Basílio*‡

‡Laboratório Associado para a Química Verde (LAQV), Rede de Química e Tecnologia (REQUIMTE), Departamento de Química, Faculdade de Ciências e Tecnologia, Universidade NOVA de Lisboa, 2829-516 Caparica, Portugal.

E-mail: nuno.basilio@fct.unl.pt

†Centro Singular de Investigación en Química Biolóxica e Materiais Moleculares (CIQUS), Departamento de Química Orgánica, Universidade de Santiago de Compostela, 15782 Santiago de Compostela, Spain.

E-mail: javier.montenegro@usc.es

## Table of Contents

1. Materials and General Methods ............ 2
2. Synthesis ............ 3
   2.1. Synthesis of the Counterion Activators ............ 3
   2.2. Synthesis of Counterion Activator 1 ............ 4
   2.3. Synthesis of Counterion Activator 2 ............ 8
   2.4. Peptide Synthesis ............ 10
3. Photochemical Characterization ............ 18
4. Binding Studies ............ 21
5. Vesicle Assays ............ 26
   5.1. LUV Preparation ............ 26
   5.2. Dye Efflux Assays ............ 26
6. Biological Assays. ............ 37
7. References ............ 41

## 1. Materials and General Methods

Commercially available reagents were used as received without further purification. All other compounds were synthesized and characterized as described below. The NMR experiments were run on a Bruker Avance III operating at 400 MHz ($^1H$) or 100 MHz ($^{13}C$). UV-Vis absorption spectra were recorded using a Varian Cary 100 Bio or a Varian Cary 5000 spectrophotometer in quartz or disposable plastic cuvettes with 10 mm optical path. Fluorescence spectra were recorded on a SPEX Fluorolog-3 Model FL3–22 spectrofluorimeter. All aqueous solutions were prepared in 5 mM phosphate buffer pH 7.4 as measured with a Crison basic 20+ pH meter.

The *E* isomers of both **1** and **2** were obtained by heating the samples at 60 ˚C overnight, while the *Z* isomers were obtained by irradiation at 366 nm until full conversions as confirmed by UV-Visible spectroscopy after the calculation of the absorption coefficients of both species. Both *E* and *Z* isomer solutions were protected from outside sources of light to avoid unwanted isomerization.

Continuous irradiations experiments were conducted in a Spex Fluorolog-2 Model F111 spectrofluorometer equipped with a 150 W Xe lamp or in a custom photochemical reactor equipped with a 200 W Hg-Xe lamp and using bandpass or cut-off filters to isolate the desired wavelengths. The light flux ($I_0$) was determined using as actinometers, ferrioxalate in water for $\lambda_{irr}$ = 365 nm ($I_0 = 3.2 \times 10^{-9}$ mol/s) and the diarylethene derivative 1,2-bis(2,4-dimethyl-5-phenyl-3-thienyl)perfluorocyclopentene in hexane for $\lambda_{irr}$ = 500 nm ($I_0 = 9.1 \times 10^{-9}$ mol/s).[1,2]

The activation energy for the thermal *Z-E* isomerization obtained by following the interconversion by UV-Vis absorption spectroscopy at three different temperatures. The first-order rate constants, $k$ ($s^{-1}$) for the interconversion at the three different temperatures were calculated and fitted to the Arrhenius equation for the obtention of the activation energy for the thermal conversion.

Confocal fluorescence microscopy images were taken with a Dragonfly spinning disc confocal microscope mounted on a Nikon Eclipse Ti-E and equipped with an Andor Zyla 4.2 PLUS sCMOS digital

camera and processed using ImageJ software (v1.52b). Flow cytometry analyses were conducted on a Guava easyCyte BG HT flow cytometer using InCyte (v3.2, GuavaSoft, Millipore). For the MTT viability assay a Tecan Infinite F200Pro plate reader was used and the curve fitting was performed with GraphPad Prism 6 software (v6.01).

# 2. Synthesis

## 2.1. Synthesis of the Counterion Activators

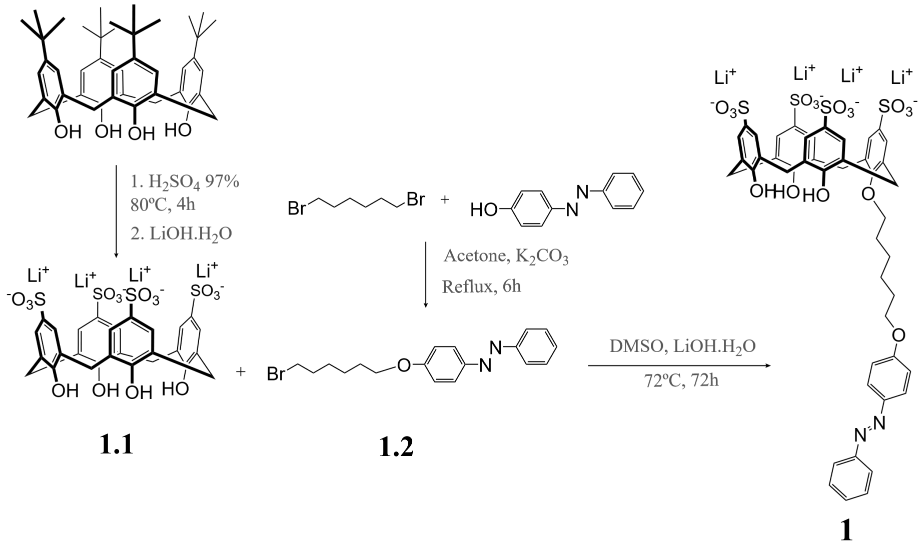


**Scheme S1** - Reaction scheme of the counterion activator, **1**, and respective precursors.

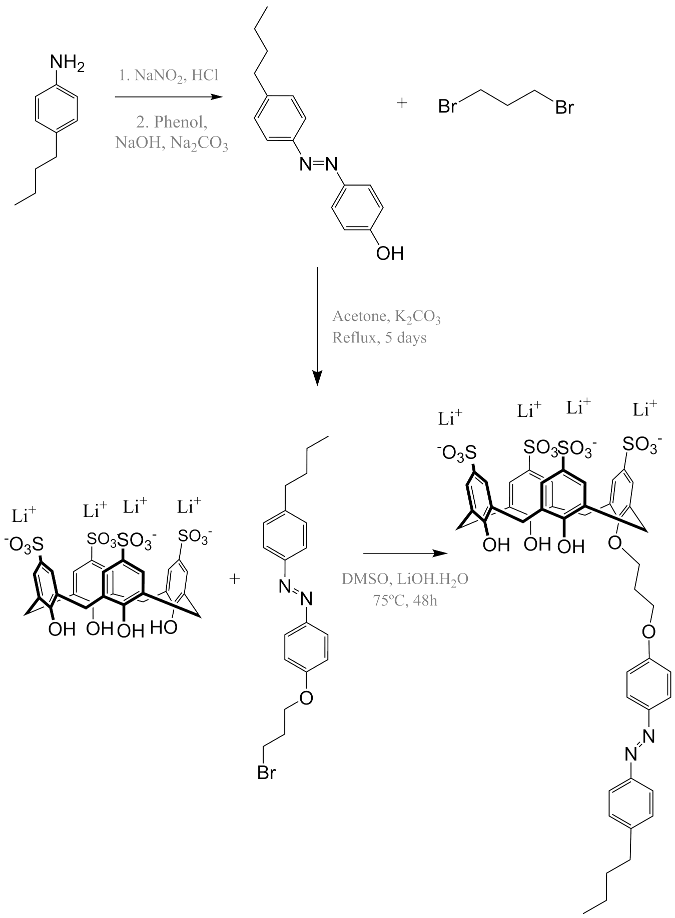


**Scheme S2** - Reaction scheme of the counterion activator, **2**, and respective precursors.

The synthesis of the precursors, **SC4**[3] **(1.1)**, **1.2**[4], **2.1**[5] and **2.2**[5] , were performed according to previously reported protocols.

## 2.2.Synthesis of Counterion Activator **1**

1.48 g (2 mmol) of compound **1.1**, 0.54 g (1.5 mmol) of compound **1.2** and 0.25 g (6 mmol) of $LiOH.H_2O$ were heated at 72 ˚C in dimethyl sulfoxide, under constant stirring, for 72h. After the reaction mixture cooled to room temperature, it was neutralized with HCl and precipitated two times, first with diethyl ether and then with ethyl acetate, in both cases not forming a precipitate but an oily mixture. The solvent was evaporated, the mixture was dissolved in methanol and precipitated with diethyl ether once again, this time forming a yellow precipitate that was filtered and dissolved in bi-distilled water. The different

by-products were separated from the compound **1** by RP-18 chromatography with 1:9 (v:v) acetonitrile:water. The fraction with the final compound was evaporated and dried under vacuum, yielding a total of 95 mg of compound **1** (6% yield). $^{1}$H NMR (400 MHz, $CD_3OD$) δ (ppm) 7.88 (t, J = 8.9 Hz, 4H, H3 and H4), 7.62 (d, J = 2.2 Hz, 2H, H13), 7.55 (d, J = 2.3 Hz, 2H, H14), 7.52 (d, J = 7.8 Hz, 2H, H2), 7.46 (t, J = 8.4, 6.0 Hz, 1H, H1), 7.38 (s, 2H, H15), 7.29 (s, 2H, H12), 7.07 (d, J = 9.0 Hz, 2H, H5), 4.64 (d, J = 12.3 Hz, 2H, H16 axial), 4.36 (d, J = 13.2 Hz, 2H, H17 axial), 4.13 (t, J = 6.5 Hz, 2H, H6), 3.90 (t, J = 6.7 Hz, 2H, H11), 3.36 (d, J = 13.3 Hz, 2H, H17 equatorial), 3.33 (d, J = 12.4 Hz, 2H, H16 equatorial), 1.92 (dq, J = 13.3, 6.6 Hz, 4H, H7 and H10), 1.68 (dq, J = 17.3, 8.2 Hz, 4H, H8 and H9). $^{13}$C NMR (101 MHz, $CD_3OD$) δ 162.06, 161.63, 158.03, 157.03, 152.77, 146.69, 138.12, 133.53, 133.21, 130.47, 130.09, 129.39, 129.21, 128.77, 128.04, 126.48, 126.02, 125.94, 125.52, 124.38, 122.09, 114.51, 75.76, 68.12, 33.39, 31.00, 29.56, 28.97, 25.78, 25.70. m/z: calculated for ($C_{46}H_{42}N_2O_{17}S_4$) $[M^{4-} + 2H^{+}]^{2-}$: 511.0688; found, 511.0691.

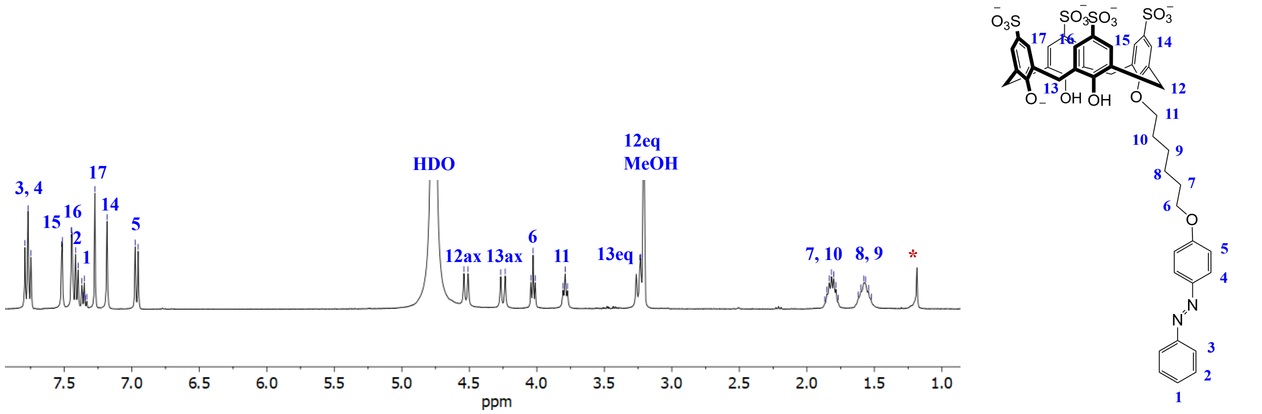


**Figure S1** - $^{1}$H NMR spectrum of compound **1** in $CD_3OD$. An impurity is marked with a red star.

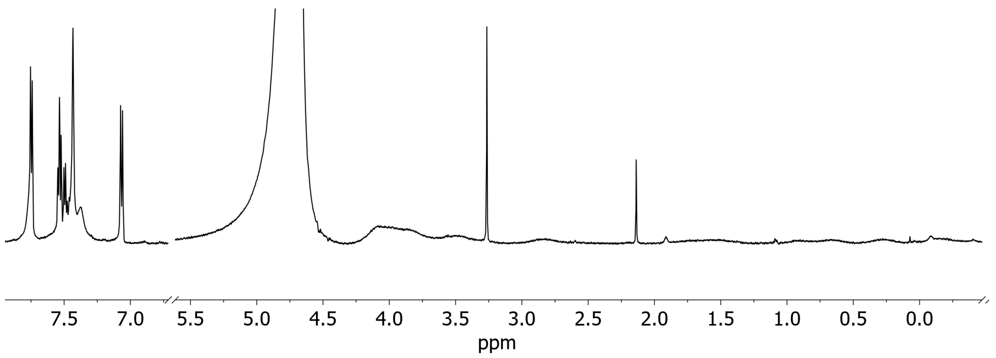


**Figure S2** - $^{1}$H NMR spectrum of compound **1** in $D_2O$.

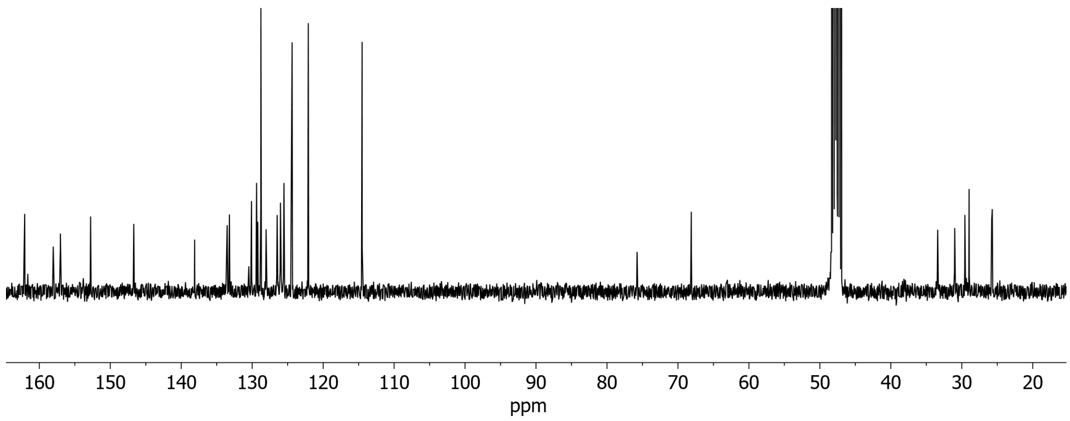


**Figure S3** - $^{13}$C NMR spectrum of compound **1** in $CD_3OD$.

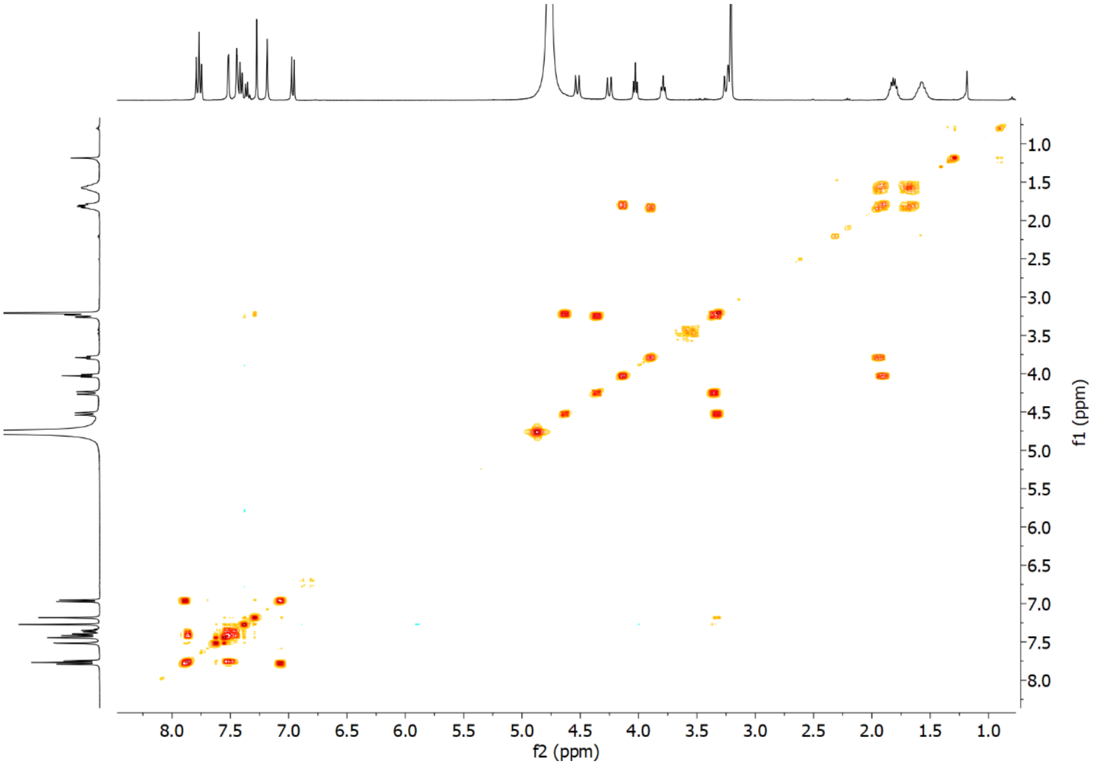


**Figure S4** - COSY spectrum of compound **1** in $CD_3OD$.

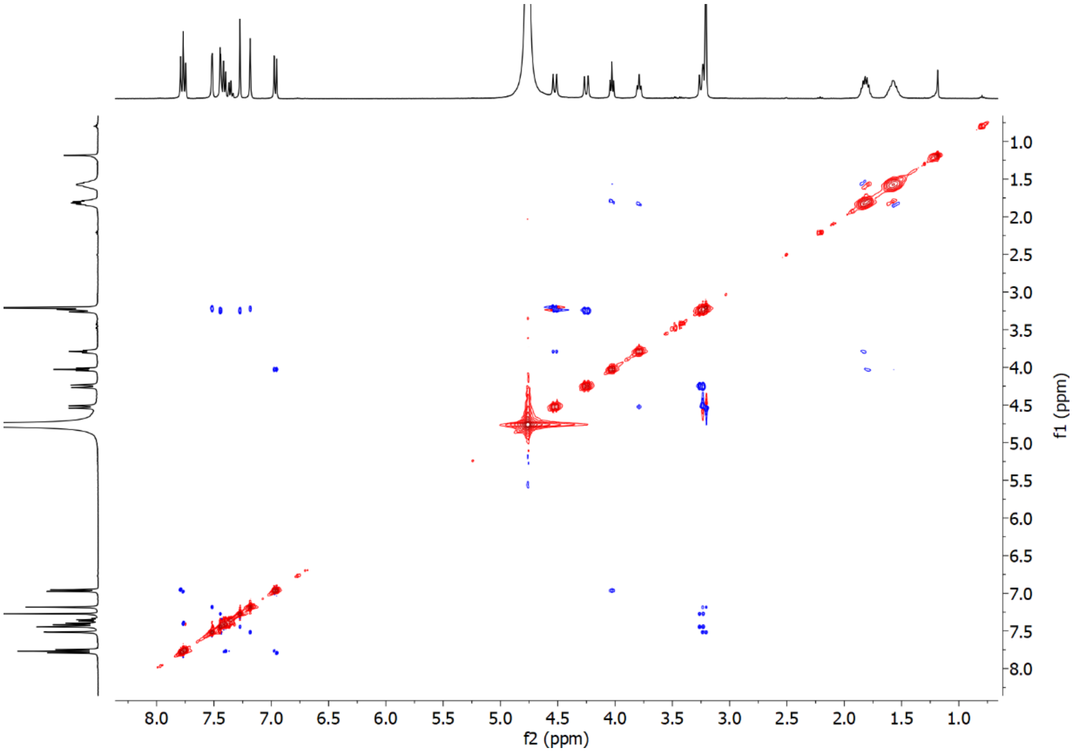


**Figure S5 -** ROESY spectrum of compound **1** in $CD_3OD$.

## **2.3.**Synthesis of Counterion Activator **2**

1.48 g (2 mmol) of **1.1**, 0.563 g (1.5 mmol) of **2.2** and 0.25 g (6 mmol) of $LiOH.H_2O$ were dissolved in 10 ml of DMSO and were heated to 75ºC under constant stirring for 48h. The reaction mixture was then precipitated two times with diethyl ether and once with ethyl acetate. The final precipitate was filtrated over vacuum, yielding a yellow powder. This was then dissolved in a small quantity of water and further purified by RP-18 chromatography, with the product being eluted with 10% acetonitrile and 90% water. After evaporation of the solvent, 277 mg of final product was obtained, corresponding to a yield of 18%. $^1$H NMR (400 MHz, $CD_3OD$) δ 7.88 (d, J = 8.7 Hz, 2H, H7), 7.79 (d, J = 8.0 Hz, 2H, H6), 7.62 (s, 2H, H15), 7.56 (d, J = 2.2 Hz, 2H, H16/H17), 7.40 (s, 2H, H16/H17), 7.34 (d, J = 8.1 Hz, 2H, H5), 7.33 (s, 2H, H14), 7.19 (d, J = 8.6 Hz, 2H, H8), 4.63 (d, J = 12.4 Hz, 2H, H12 axial), 4.60 (t, J = 14.3 Hz, 2H, H9), 4.38 (d, J = 13.2 Hz, 2H, H13 axial), 4.06 (t, J = 5.5 Hz, 1H), 3.37 (d, J = 15.0 Hz, 2H, H13 equatorial), 3.34 (d, J = 31.1 Hz, 2H, H12 equatorial), 2.71 (t, J = 7.7 Hz, 2H, H4), 2.45 (d, J = 8.6 Hz, 2H, H10), 1.68 (p, J = 7.5 Hz, 2H, H3), 1.42 (h, J = 7.5 Hz, 2H, H2), 0.99 (t, J = 7.3 Hz, 3H, H1). $^{13}$C NMR (101 MHz, $CD_3OD$) δ 161.78, 157.01, 151.02, 146.79, 145.65, 133.58, 133.26, 129.38, 129.09, 128.72, 128.09, 126.47, 126.02, 125.50, 124.19, 122.14, 114.69, 65.23, 35.08, 33.39, 30.92, 29.63, 21.98, 12.86. HRMS-ESI (negative mode) m/z: calculated for $(C_{47}H_{45}N_2O_{17}S_4)$ $[M^{4-} + 3H^+]^-$: 1037.1606; found, 1037.1591.

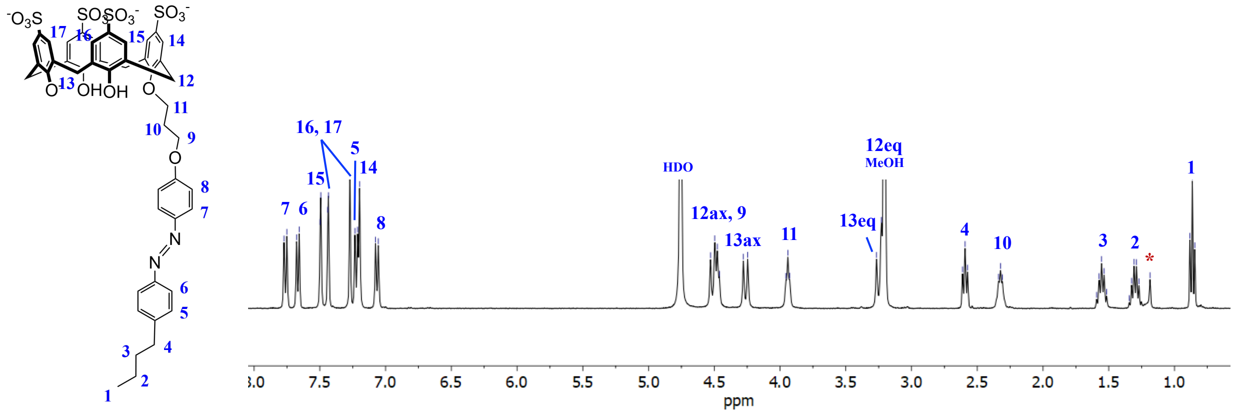


**Figure S6** - $^1$H NMR spectrum of compound **2** in $CD_3OD$. Impurity is marked with a red star.

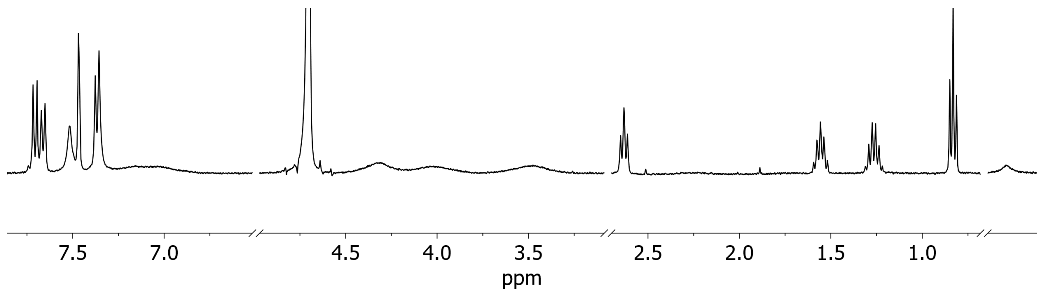


**Figure S7** - $^{1}$H NMR spectrum of compound **2** in $D_2O$.

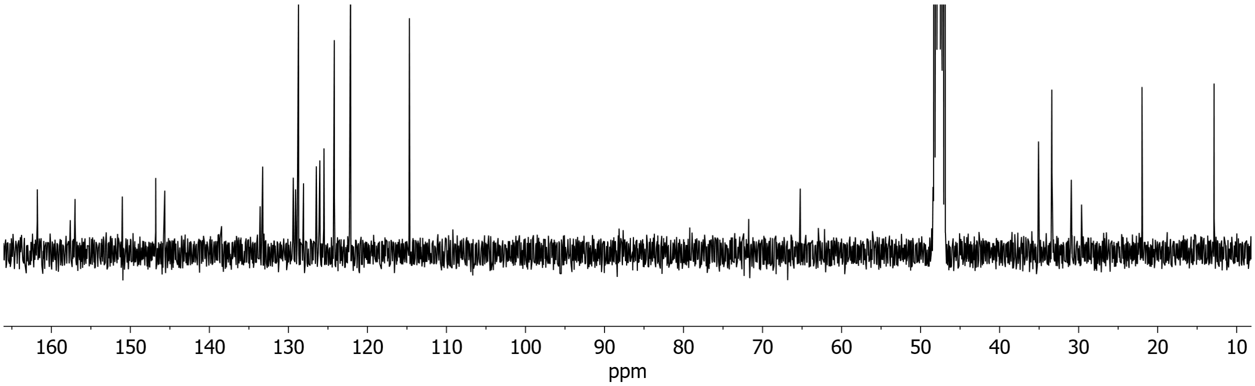


**Figure S8** - $^{13}$C NMR spectrum of compound **2** in $CD_3OD$.

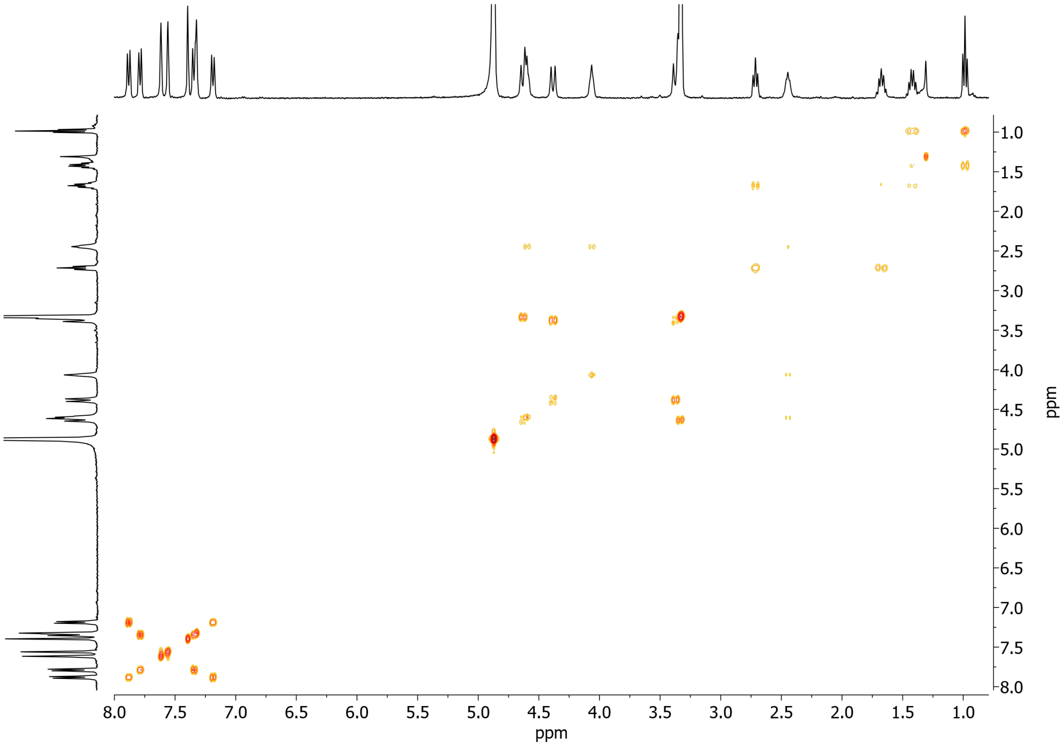


**Figure S9 -** COSY spectrum of compound **2** in $CD_3OD$.

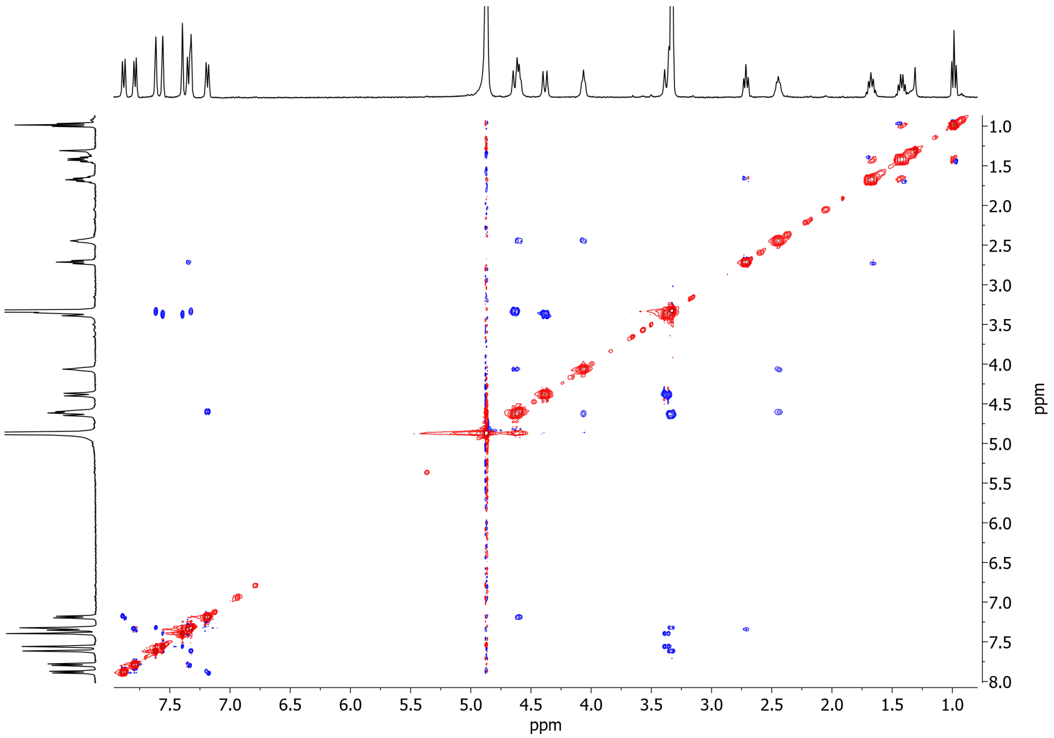


**Figure S10** - ROESY spectrum of compound **2** in $CD_3OD$.

## 2.4.Peptide Synthesis

Peptide synthesis was performed following the manual Fmoc solid-phase peptide synthesis (SPPS) using Rink Amide resin (loading 0.45 mmol/g).[6] Firstly, the resin was placed in a peptide synthesis vessel and swelled in DMF (3 ml) bubbling Ar for 20 min. Peptide grown was performed by successive cycles of deprotection and coupling cycles. Deprotection steps consisted in the *N* terminus Fmoc deprotection, accomplished by bubbling 20% piperidine in DMF (3 ml, 15 min), filtering it and carrying out final washes with DMF (3 x 3 ml, 5 min). Coupling cycles were carried out by treatment with a solution of the corresponding Fmoc-protected α-amino acid (3 equiv), *N*-HBTU (2.8 equiv) and DIEA (4 equiv) in DMF (3 ml, 15 min) and the resin was washed with DMF (3 x 3 ml, 5 min). Alternatively, coupling of the TAMRA fluorophore was accomplished by the addition of a solution of 5-Carboxytetramethylrhodamine (1 equiv), HATU (1 equiv) and DIEA (0.195 M, 1 equiv) in DMF (2 mL) bubbling Ar for 4 hours.

Then, peptides were deprotected, sequentially washed with DMF (3 x 3 ml, 5 min) followed by DCM (3 x 3 ml, 5 min), and cleaved from the resin by standard TFA cleavage procedure at rt using a TFA:DCM:TIS:$H_2O$ solution (90:5:2.5:2.5, 3 ml, 2 h). Afterwards, the mixture was filtered, washed with TFA (2 x 1 ml) and peptides were precipitated in ice-cold $Et_2O$ (25 ml). Precipitates were centrifuged and dissolved in $H_2O$ (5 ml) to carry on with the purification by reverse phase chromatography with a gradient from 5:95 ($CH_3CN$ (0.1% TFA):$H_2O$ (0.1% TFA)) to 95:5 ($CH_3CN$ (0.1% TFA):$H_2O$ (0.1% TFA)) in 35 min (after first 5 min with isocratic flow in conditions 5:95 ($CH_3CN$ (0.1% TFA):$H_2O$ (0.1% TFA))). Finally, the collected fractions were lyophilised and stored at -20 °C. Purity and identity were confirmed by analytical HPLC and mass spectrometry.

2.4.1. Synthesis of peptide $R_4$

Peptide $R_4$ (Figure S11) was prepared following the previous general procedure for peptide synthesis with an overall yield of 27%. **ESI-HPLC**: (Agilent SB-C18 column, $H_2O$ (0.1% TFA)/$CH_3CN$ (0.1% TFA) 95:5 (0→2 min), 95:5→5:95 (2→21 min), 5:95 (>21 min), ESI, +*e*V). R*t* = 5.87 min. m/z = 642.5 (14, $[M+H]^+$), 378.8 (10, $[M+2H+TFA]^{2+}$), 321.8 (100, $[M+2H]^{2+}$), 214.9 (23, $[M+3H]^{3+}$).

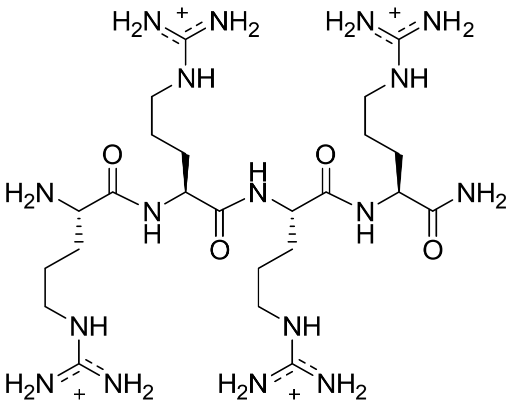


**Figure S11** - Structure of the 4 residue oligoarginine, $R_4$.

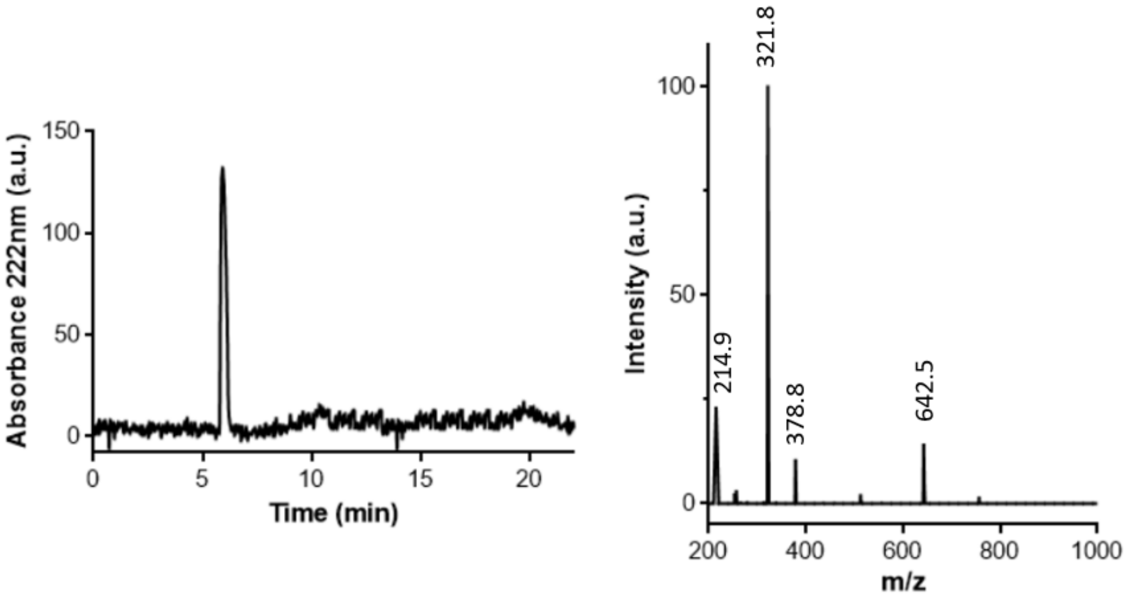


**Figure S12** - HPLC chromatogram for peptide $R_4$ and respective ESI-MS spectrum.

2.4.2. Synthesis of peptide $R_6$

Peptide $R_6$ (Figure S13) was prepared following the previous general procedure for peptide synthesis with an overall yield of 55%. **ESI-HPLC**: (Agilent SB-C18 column, $H_2O$ (0.1% TFA)/$CH_3CN$ (0.1% TFA) 95:5 (0→2 min), 95:5→5:95 (2→21 min), 5:95 (>21 min), ESI, +*e*V). R*t* = 6.86 min. m/z = 592.0 (13, $[M+2H+2TFA]^{2+}$), 534.9 (56, $[M+2H+TFA]^{2+}$), 477.9 (48, $[M+2H]^{2+}$), 395.0 (12, $[M+3H+2TFA]^{3+}$), 357.0 (32, $[M+3H+TFA]^{3+}$), 319.1 (100, $[M+3H]^{3+}$), 239.6 (16, $[M+4H]^{4+}$).

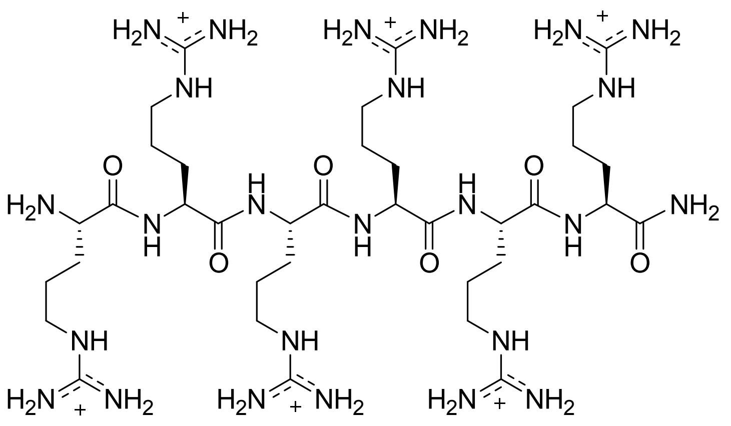

**Figure S13** - Structure of the 6 residue oligoarginine, $R_6$.

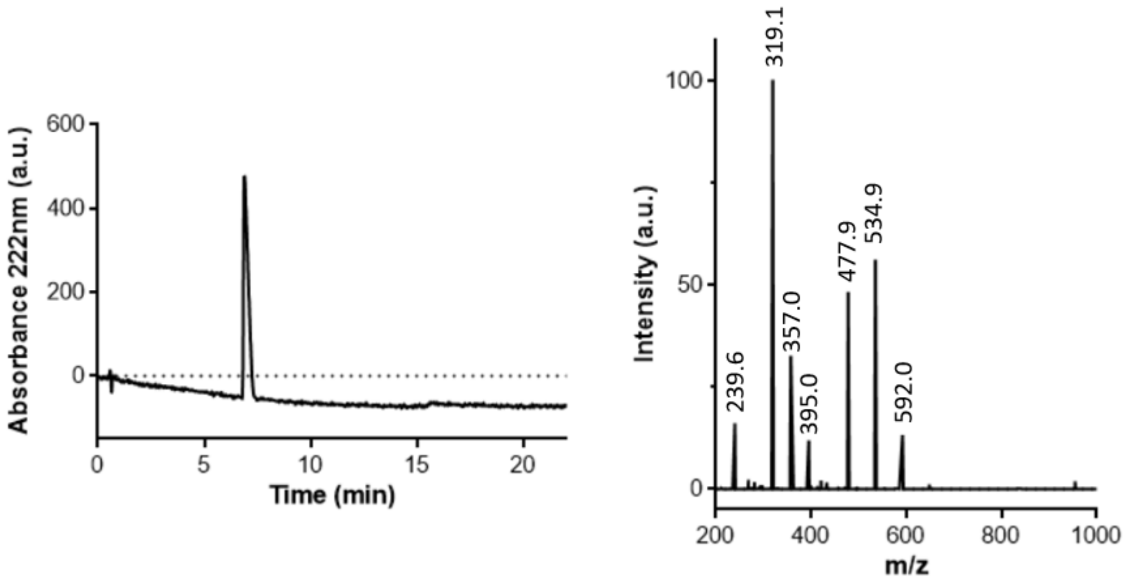


**Figure S14** - HPLC chromatogram for peptide $R_6$ and respective ESI-MS spectrum.

### 2.4.3. Synthesis of peptide $R_8$

Peptide $R_8$ (Figure S15) was prepared following the previous general procedure for peptide synthesis with an overall yield of 37%. **ESI-HPLC:** (Agilent SB-C18 column, $H_2O$ (0.1% TFA)/$CH_3CN$ (0.1% TFA) 95:5 (0→2 min), 95:5→5:95 (2→21 min), 5:95 (>21 min), ESI, +*e*V). R*t* = 5.23 min. m/z = 861.9 (17, $[M+2H+4TFA]^{2+}$), 805.0 (27, $[M+2H+3TFA]^{2+}$), 748.0 (28, $[M+2H+2TFA]^{2+}$), 691.0 (19, $[M+2H+TFA]^{2+}$), 575.2 (23, $[M+3H+4TFA]^{3+}$), 537.2 (79, $[M+3H+3TFA]^{3+}$), 499.1 (100, $[M+3H+2TFA]^{3+}$), 461.1 (70, $[M+3H+TFA]^{3+}$), 423.1 (54, $[M+3H]^{3+}$), 374.6 (7, $[M+4H+2TFA]^{4+}$), 346.1 (15, $[M+4H+TFA]^{4+}$), 317.7 (34, $[M+4H]^{4+}$).

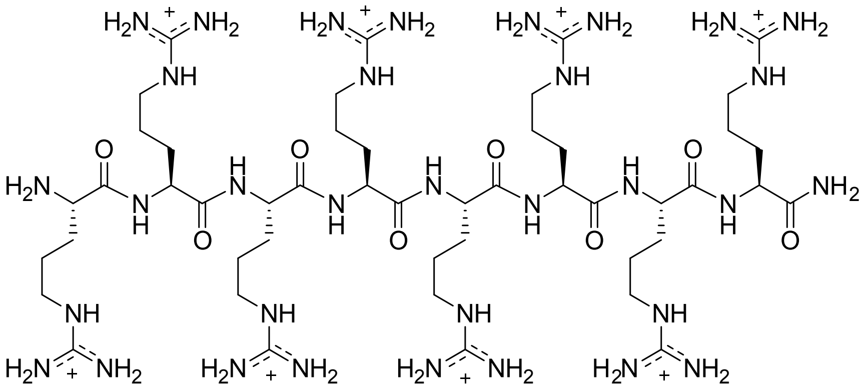

**Figure S15** - Structure of the 8 residue oligoarginine, $R_8$.

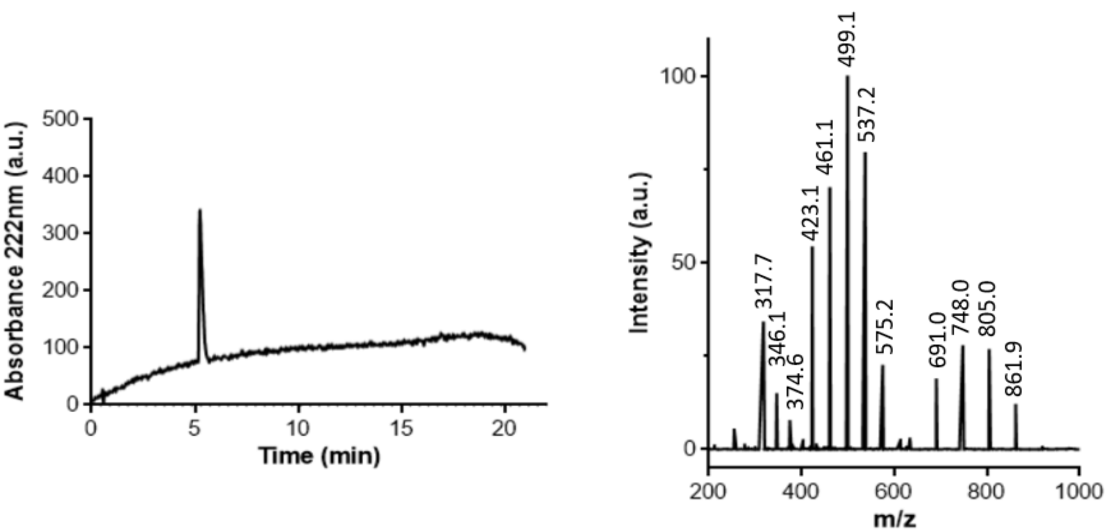


**Figure S16** - HPLC chromatogram for peptide $R_8$ and respective ESI-MS spectrum.

2.4.4. Synthesis of peptide $E_6$

Peptide $E_6$ (Figure S17) was prepared following the previous general procedure for peptide synthesis with an overall yield of 14%. **ESI-HPLC:** (Agilent SB-C18 column, $H_2O$ (0.1% TFA)/$CH_3CN$ (0.1% TFA) 100:0→25:75 (0→21 min), ESI, +*e*V). R*t* = 4.38 min. m/z = 396.8 (85, $[M+2H]^{2+}$), 792.3 (100, $[M+H]^{+}$).

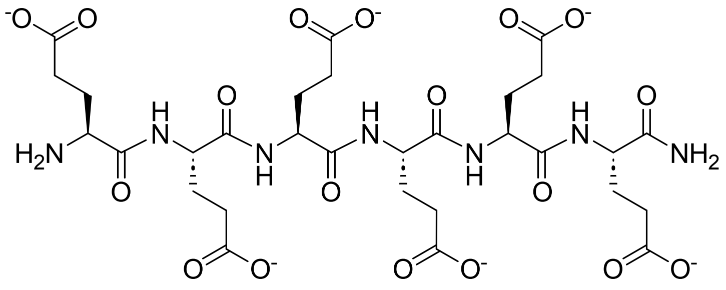

**Figure S17** - Structure of the 6 residue oligoglutamic, $E_6$.

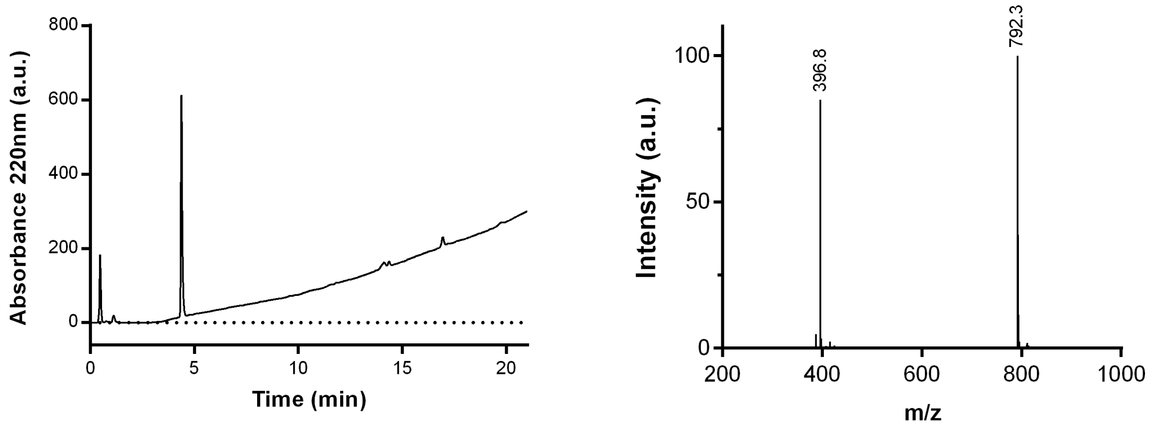


**Figure S18** - HPLC chromatogram for peptide $E_6$ and respective ESI-MS spectrum.

### 2.4.5. Synthesis of peptide $E_8$

Peptide $E_8$ (Figure S19) was prepared following the previous general procedure for peptide synthesis with an overall yield of 9%. **ESI-HPLC:** (Agilent SB-C18 column, $H_2O$ (0.1% TFA)/$CH_3CN$ (0.1% TFA) 75:25→25:75 (0→21 min), ESI, +*e*V). R*t* = 5.26 min. m/z = 525.8 (100, $[M+2H]^{2+}$), 1050.4 (54, $[M+H]^{+}$).

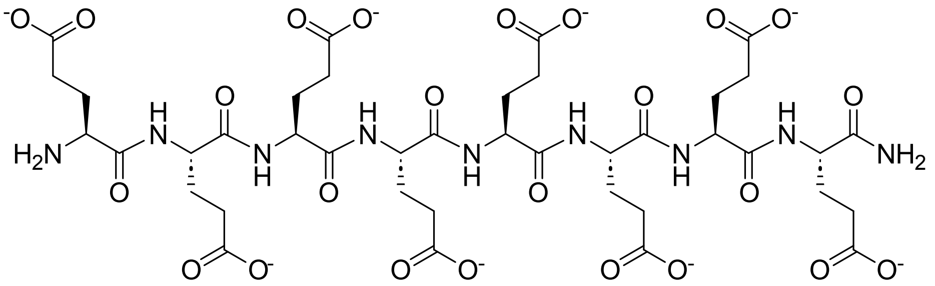


**Figure S19** - Structure of the 6 residue oligoglutamic, $E_8$.

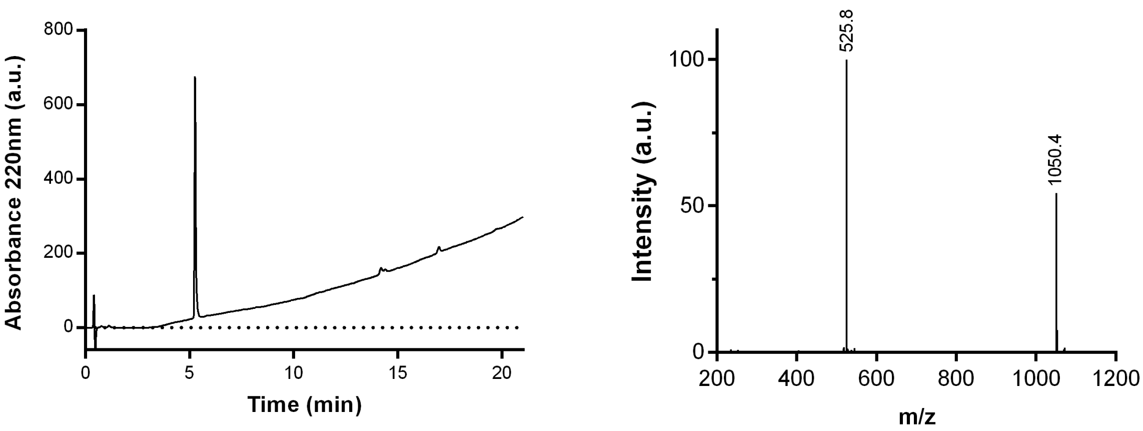


**Figure S20** - HPLC chromatogram for peptide $E_8$ and respective ESI-MS spectrum.

2.4.6. Synthesis of peptide TAMRA-$R_8$

Peptide TAMRA-$R_8$ (Figure S21) was prepared following the previous general procedure for peptide synthesis with an overall yield similar as previously reported.[8] **ESI-HPLC:** (Agilent SB-C18 column, $H_2O$ (0.1% TFA)/$CH_3CN$ (0.1% TFA) 95:5→5:95 (0→12 min), ESI, +*e*V). R*t* = 5.96 min. m/z = 712.4 (37, $[M+3H+3TFA]^{3+}$), 674.2 (100, $[M+3H+2TFA]^{3+}$), 636.3 (98, $[M+3H+TFA]^{3+}$), 598.2 (34, $[M+3H]^{3+}$), 534.5 (21, $[M+4H+3TFA]^{4+}$), 506.0 (32, $[M+4H+2TFA]^{4+}$), 477.5 (41, $[M+4H+TFA]^{4+}$), 449.1 (51, $[M+4H]^{4+}$), 382.3 (11, $[M+5H+TFA]^{5+}$), 359.5 (29, $[M+5H]^{5+}$).

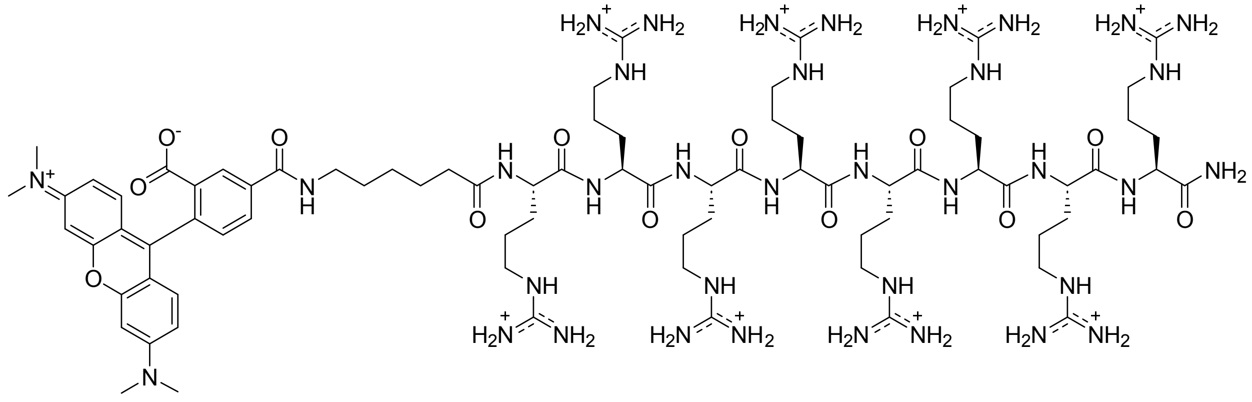

**Figure S21** - Structure of the 8 residue oligoarginine labelled with TAMRA fluorophore, TAMRA-$R_8$.

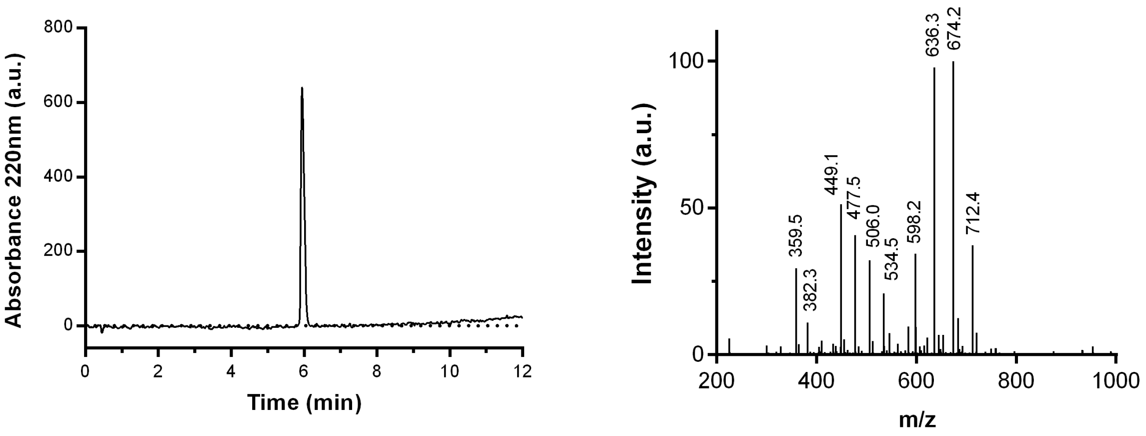


**Figure S22** - HPLC chromatogram for peptide TAMRA-$R_8$ and respective ESI-MS spectrum.

# 3. Photochemical Characterization

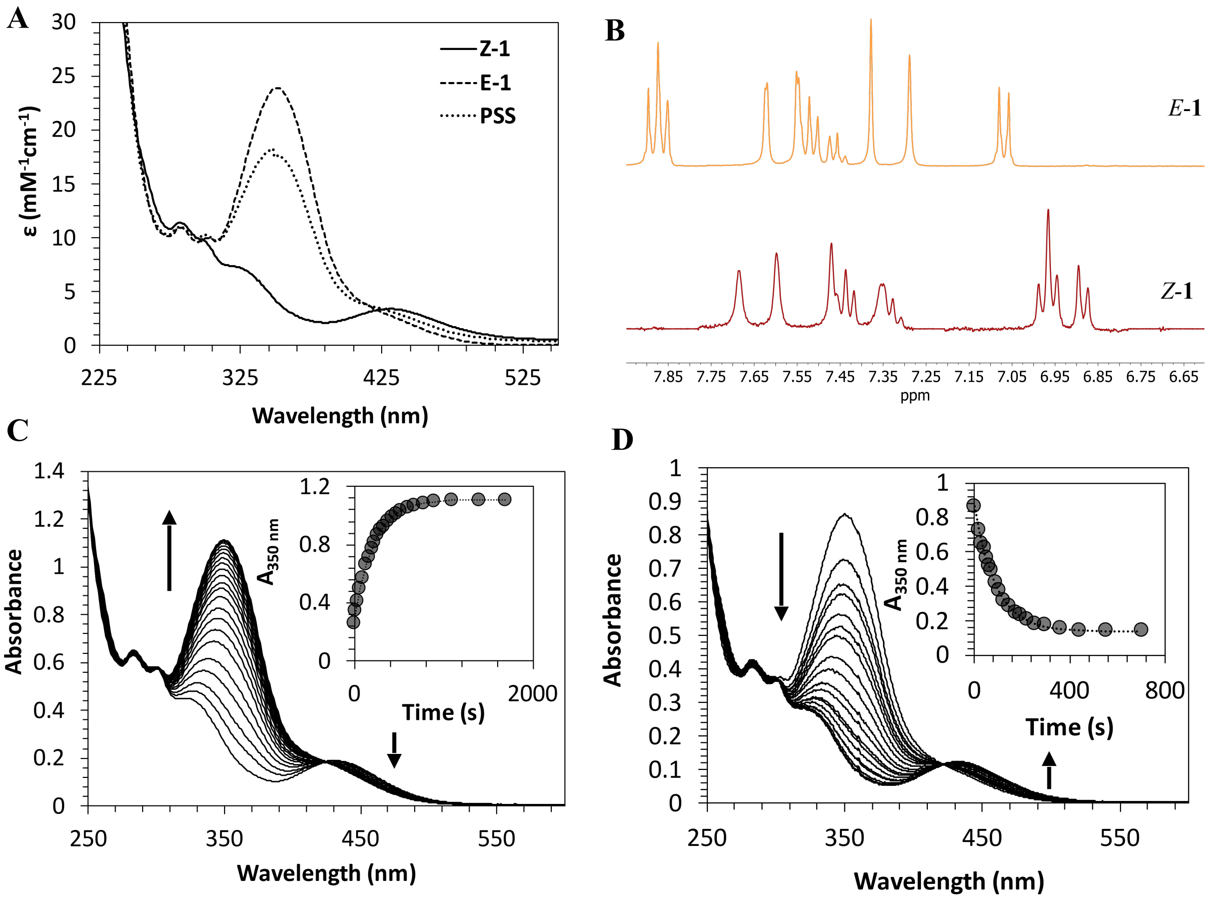


**Figure S23** - A) Absorbance spectra of both *E* and *Z* isomers of **1** and the photostationary state (PSS; 70:30 of *E:Z*) obtained after irradiation at 500 nm; B) Partial $^1$H NMR (in $CD_3OD$) spectra of the aromatic region of the **1** at the dark-adapted state (*E*-isomer) and after irradiation at366 nm (*Z*-isomer). C) *Z*→*E* and D) *E*→*Z* photoisomerization of **1** followed by UV-Vis spectroscopy upon irradiation at 500 nm and 366 nm, respectively.

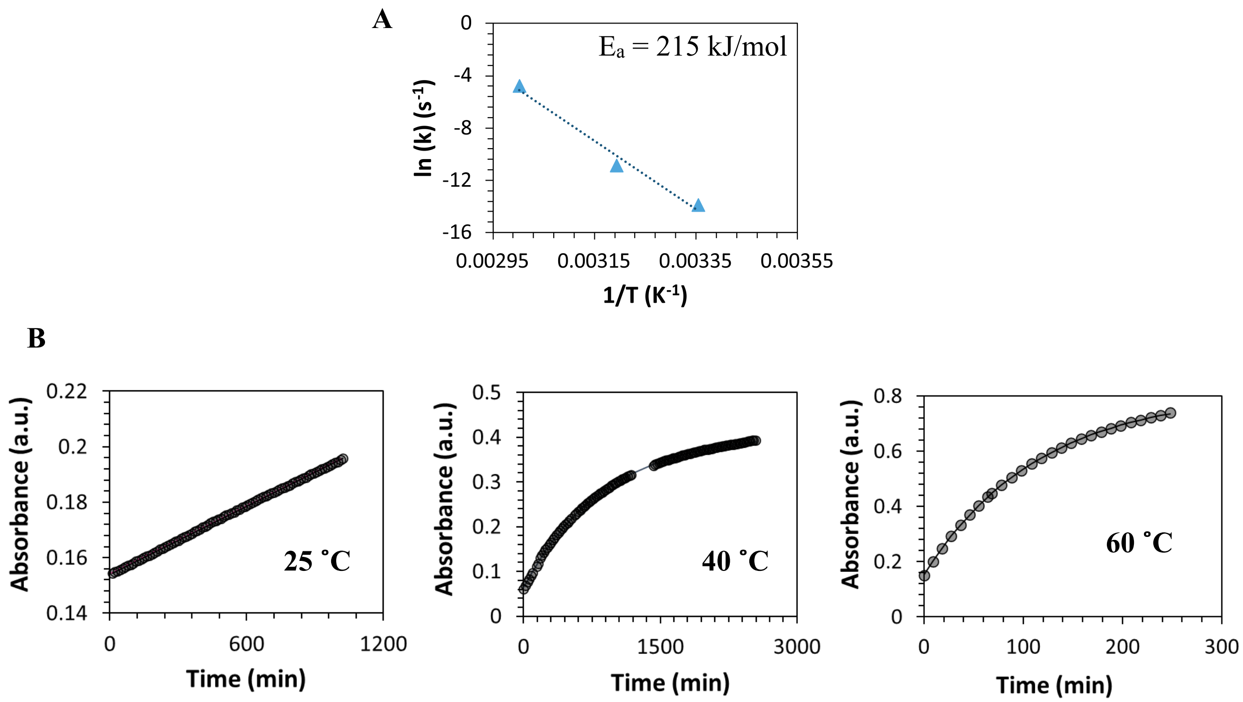


**Figure S24** - A) Linearization of the rate constant of *Z*-**1** thermal conversion to the *E* isomer, in function of the temperature. B) Thermal conversion of *Z*-**1** to *E*-**1** at different temperatures, used for the calculation of the activation energy of the thermal isomerization. The rate constants calculated for the conversion at 25, 40 and 60 ˚C were of $1.03\times10^{-6}$, $1.87\times10^{-5}$ and $8.68\times10^{-3}$ $s^{-1}$, respectively.

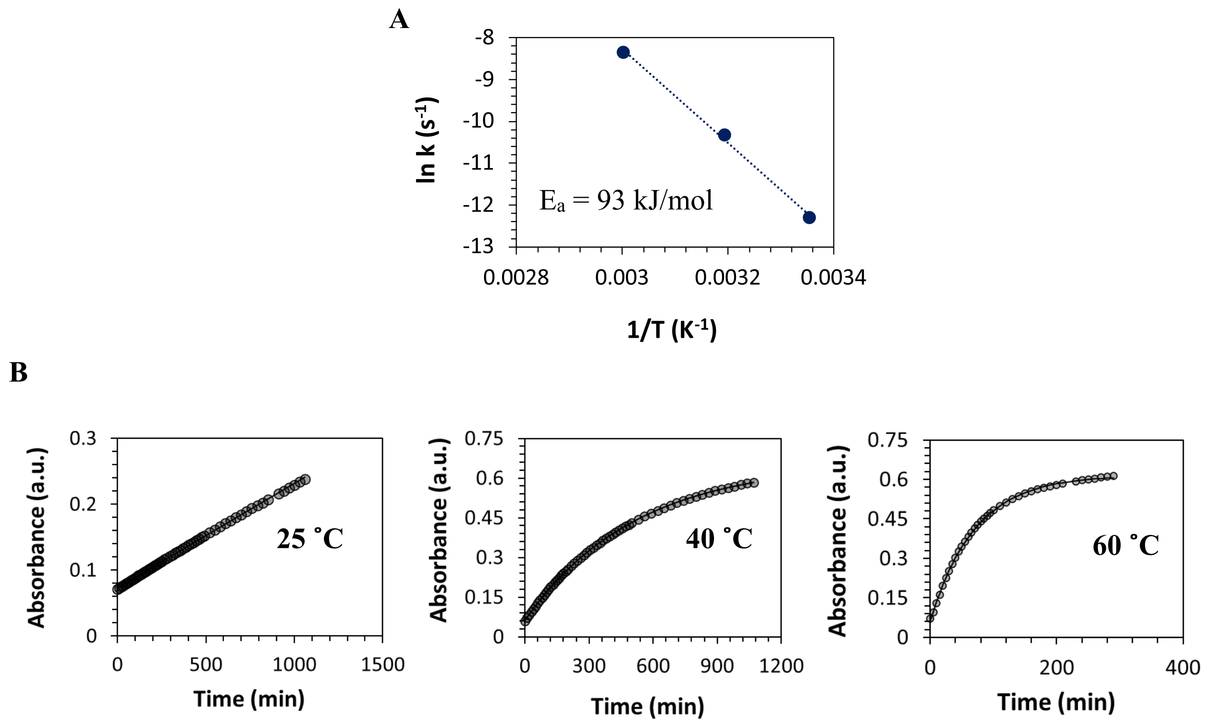


**Figure S25** - A) Linearization of the rate constant of *Z*-**2** thermal conversion to the *E* isomer, in function of the temperature. B) Thermal conversion of *Z*-**2** to *E*-**2** at different temperatures, used for the calculation of the activation energy of the thermal isomerization. The rate constants calculated for the conversion at 25, 40 and 60 ˚C were of $4.55\times10^{-6}$, $3.24\times10^{-5}$ and $2.37\times10^{-4}$ $s^{-1}$, respectively.

## 4. Binding Studies

The binding affinities of the counterion activators and peptide complexes were analyzed by indicator displacement assays. The fluorescent dye chosen for these experiments was lucigenin (LCG) due to its complex with the receptor SC4 being well characterized and due to the high variation of emission between its bound and free form. Firstly, the affinity for LCG was calculated for each of the activators, by titrations followed by fluorescence spectroscopy leading to almost full quenching of the emission. For the competitive assays, a concentration of LCG and activator was chosen in order to have approximately 90% of complexed dye and that mixture was then titrated with each of the peptides, leading to the regaining of emission when LCG was release from the complex.

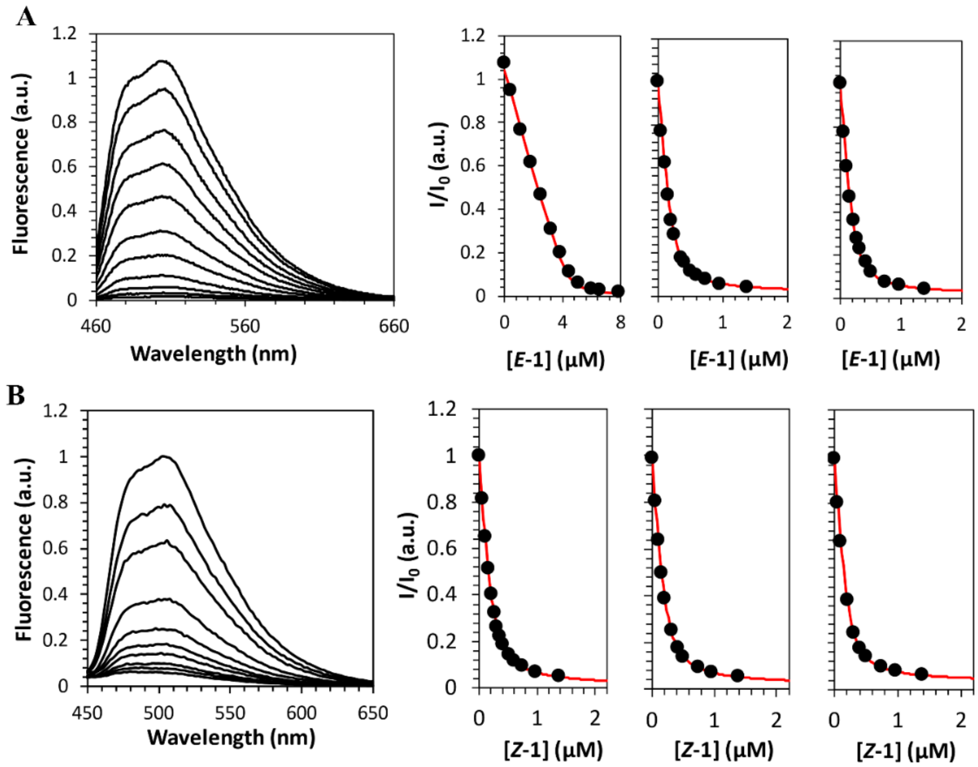


**Figure S26 -** Fluorescence titration of the lucigenin dye with increasing concentrations of A) *E*-**1** and B) *Z*–**1** in water (5 mM phosphate buffer pH 7.3). LCG concentration was fixed 0.2 µM in all experiments, except for the first *E*-**1** titrations were [LCG] = 4.8 µM.

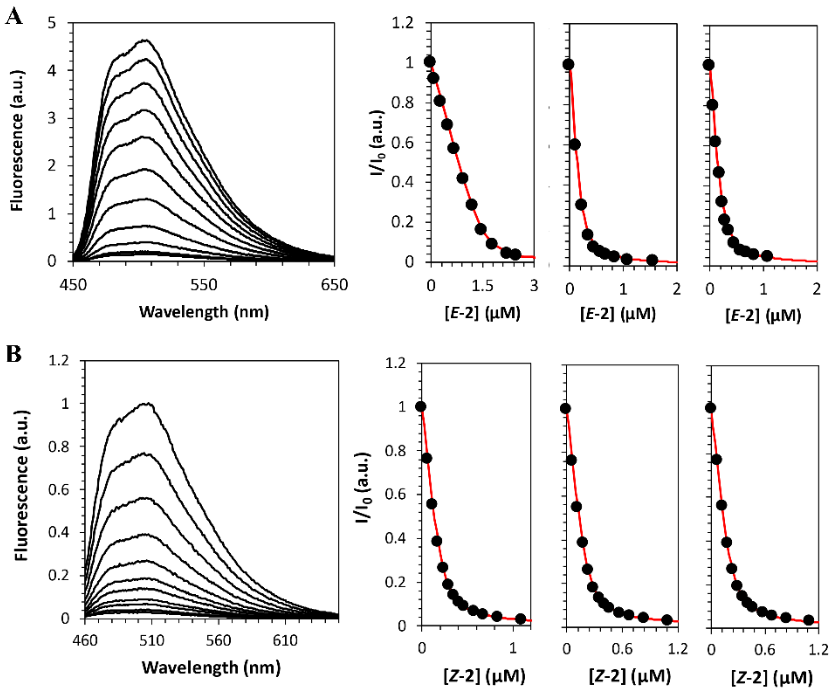


**Figure S27** - Fluorescence titrations of the lucigenin dye (LCG, 0.2 µM, except for the first *E*-**2** titration were [LCG] = 1.5 µM ) with increasing concentrations of (A) *E*-**2** and (B) *Z*-**2** in water (5 mM phosphate buffer pH 7.3). All titrations were performed three times.

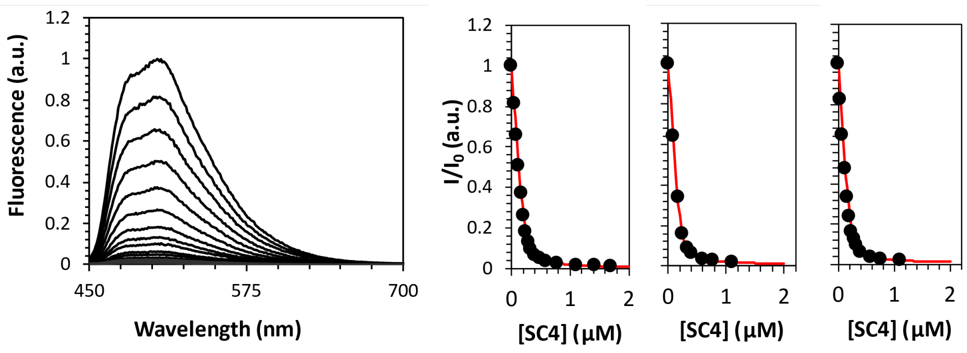


**Figure S28** - Fluorescence titrations of the lucigenin dye (LCG, 0.2 µM) with increasing concentrations of SC4 in water (5 mM phosphate buffer pH 7.3). The titration was performed three times.

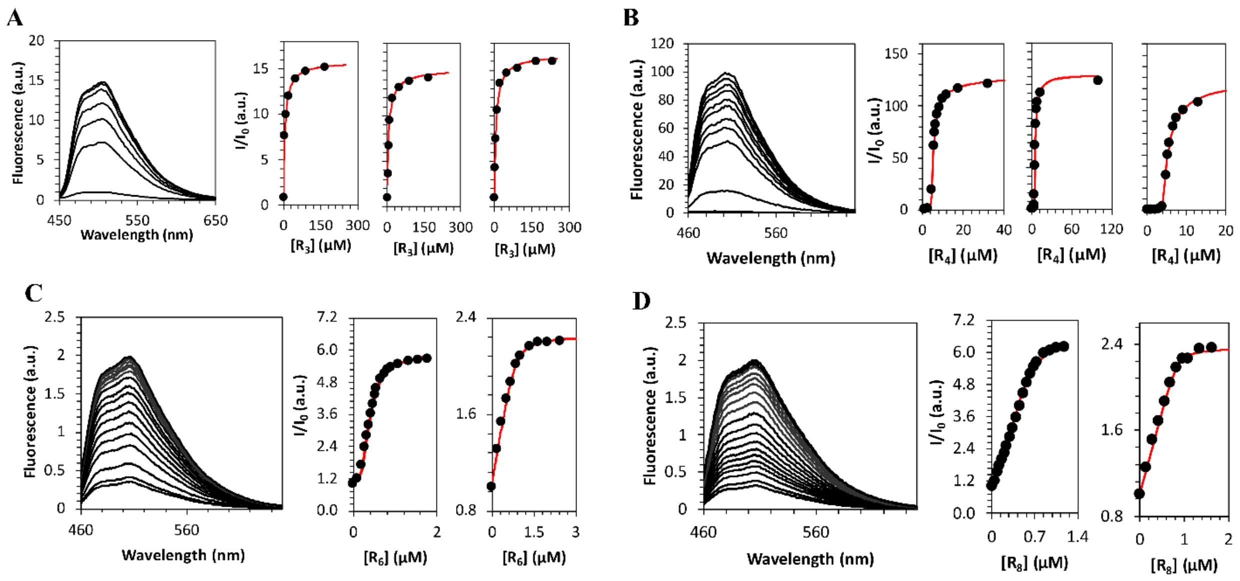


**Figure S29** - Indicator displacement titration of the several oligoarginines A) $R_3$ B) $R_4$ C) $R_6$ and D) $R_8$, using SC4:LCG as the reporter pair (5 mM phosphate buffer pH 7.3). LCG and SC4 concentrations were fixed at A) 0.22 and 0.50 µM, for all three replicates, B) 0.22 and 4.7 µM for the first titration and 0.22 and 4.3 µM for the remaining two replicates, C) 0.22 and 0.45 µM and 1.1 and 0.85 µM and D) 0.28 and 0.43 µM and 1.1 and 0.85 µM, respectively, from left to right.

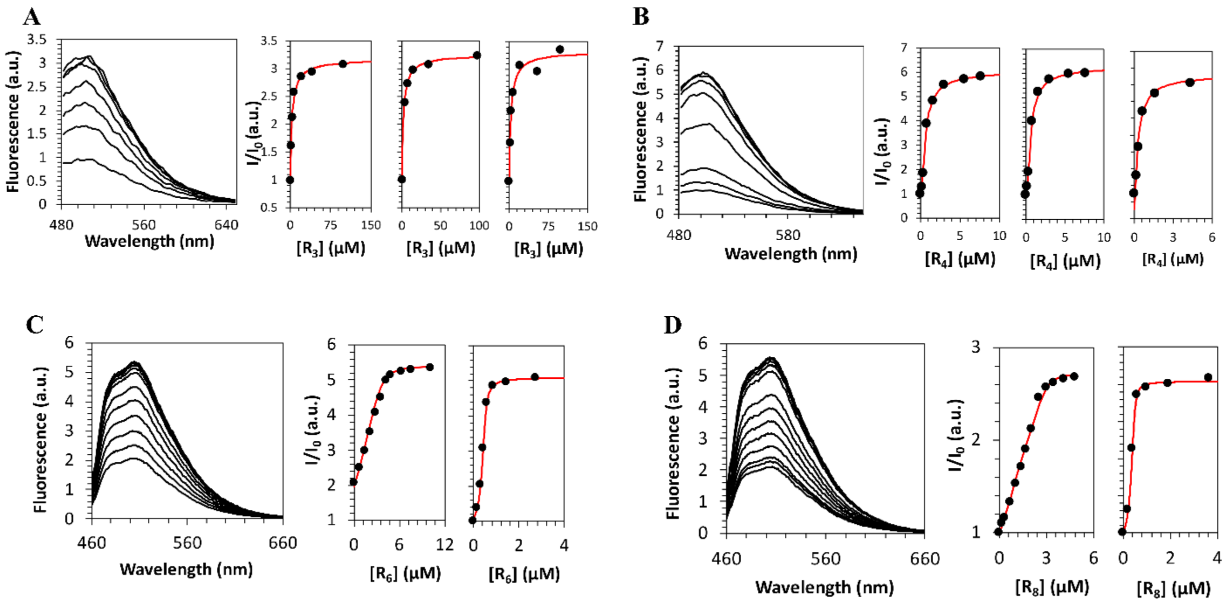


**Figure S30** - Indicator displacement titration of the several oligoarginines A) $R_3$ B) $R_4$ C) $R_6$ and D) $R_8$, using *E*-**1**:LCG as the reporter pair (5 mM phosphate buffer pH 7.3) LCG and *E*-**1** concentrations were fixed at A) 0.11 and 0.25 µM, for all three replicates, B) 0.22 and 0.5 µM, for the first two titrations and 0.22 and 0.23 µM for the remaining replicate, C) 4.0 and 3.8 µM and 0.22 and 0.5 µM, D) 4 and 3 µM and 0.22 and 0.5 µM, respectively, from left to right.

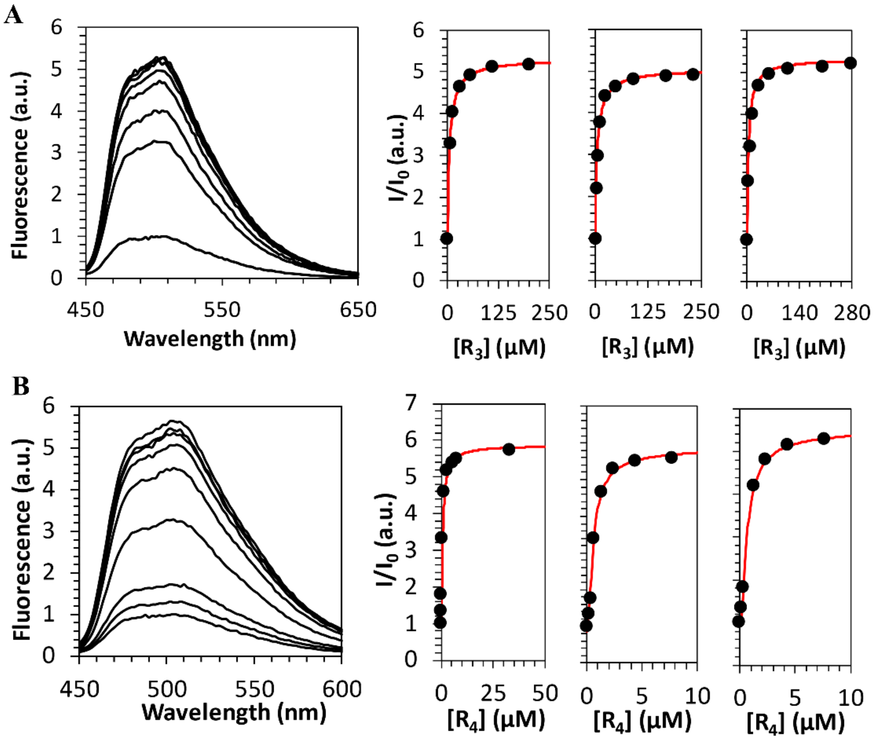


**Figure S31** - Indicator displacement titration of the oligoarginines A) $R_3$ and B) $R_4$, using *Z*-**1**:LCG as the reporter pair (5 mM phosphate buffer pH 7.3) LCG and *Z*-**1** concentrations were fixed at 0.22 and 0.5 μM, respectively, for all experiments.

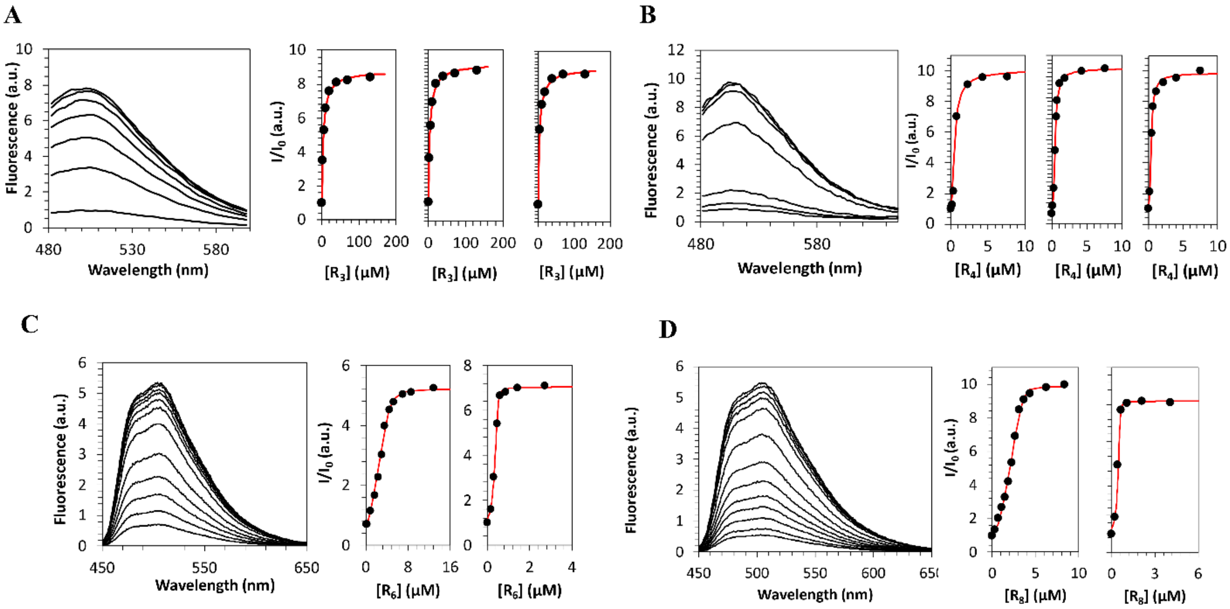


**Figure S32** - Indicator displacement titration of the several oligoarginines A) $R_3$ B) $R_4$ C) $R_6$ and D) $R_8$, using *E*-**2**:LCG as the reporter pair (5 mM phosphate buffer pH 7.3) LCG and *E*-**2** concentrations were fixed at A) 0.2 and 0.6 μM, for the first two titrations, and 0.22 and 0.6 μM, for the remaining replicate, B) 0.22 and 0.5 μM, 0.22 and 0.58 μM and 0.22 and 0.55 μM, C) 4 and 4.3 μM and 0.22 and 0.5 μM and D) 3.6 and 4.3 μM and 0.22 and 0.6 μM, respectively, from left to right.

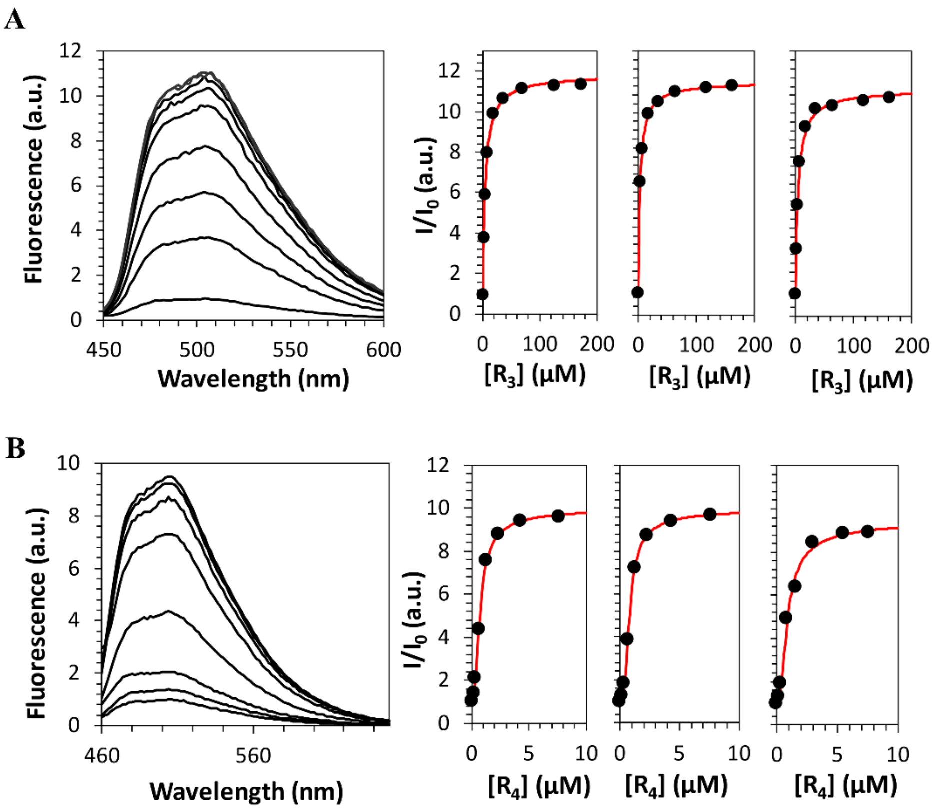


**Figure S33** - Indicator displacement titration of the oligoarginines A) $R_3$ and B) $R_4$, using *Z*-**2**:LCG as the reporter pair (5 mM phosphate buffer pH 7.3) LCG and *Z*-**2** concentrations were fixed at A) 0.22 and 0.5 µM, for the three replicates, and B) 0.22 and 0.66 µM, 0.22 and 0.72 µM and 0.22 and 0.77 µM, respectively, from left to right.

## 5. Vesicle Assays

### 5.1. LUV Preparation

10 mg of L-α-phosphatidylcholine (95%) (Egg, Chicken) were dissolved in 1 ml of chloroform. The solution was evaporated under a nitrogen stream, under rotation, to form a thin lipid film in the bottom of the flask. This film was left to dry under vacuum for a minimum of 1h. After drying the maximum of the solvent from the solution, 1 ml of the solution to incorporate (CF 50 mM in 5 mM phosphate buffer for EYPC-LUV⊃CF and only phosphate buffer 5 mM pH 7.4, for the LUVs with no encapsulation) was added to the thin film and the lipids were left to resuspend under agitation, in a 40˚C bath, for 30 min. The resuspended lipid mixture was then submitted to 10 freeze-thaw cycles and extruded 23 times through a 100 nm membrane, to obtain uniform, monodisperse liposomes with high encapsulation of the added solution. The liposomes were then separated from the molecules that were not encapsulated by size-exclusion chromatography, with Sephadex G25 (V = 37 ml), obtaining two fractions, one with high turbidity and a clear one, corresponding to the encapsulated and free sensor, respectively. The samples were then analyzed by Dynamic Light Scattering (DLS) to determine the average size of the liposomes in solution and their size distribution.

All assays were done up to 48h after the preparation of the vesicles to ensure their integrity and majority of CF still encapsulated.

### 5.2. Dye Efflux Assays

**$EC_{50}$ and $Y_{max}$ calculation**. For the measurement of **1** and **2**'s effectiveness as a counterion activators of polyarginine transport, dye efflux assays were executed at several counterion activator concentrations, while maintaining the concentration of peptide and LUV fixed as indicated in the respective figures.

For the dye efflux assays, CF emission was followed throughout the assay, with consecutive additions of the elements of the solution – the initial solution presented only the vesicles at a fixed concentration. A counterion activator aliquot is then added according to the concentration to be tested and the emission is measured to confirm that CF remains encapsulated; some counterion activators can act as detergents at sufficient concentrations. Finally, the peptide in question is added and fluorescence spectra are measured

until the emission stabilizes and a maximum of release is reached. If the counterion activator has the capacity to activate the transport, CF should be co-transported to the exterior of the liposome with the influx of the counterion-peptide complex. Finally, Triton X-100 is added to the solution to dissolve the vesicles and obtain the maximum amount of CF that could be released from the liposomes and emit in the outside medium.

$EC_{50}$ and $Y_{max}$ values were calculated from the values of corrected emission at the plateau that is reached, according to previously reported assays.[7]

**Light-activated release assays.** The light-modulation offered by this novel counterion activator was tested by performing dye efflux assays as previously described, with the addition of an interval in the measurement where the solution was irradiated at 500 nm.

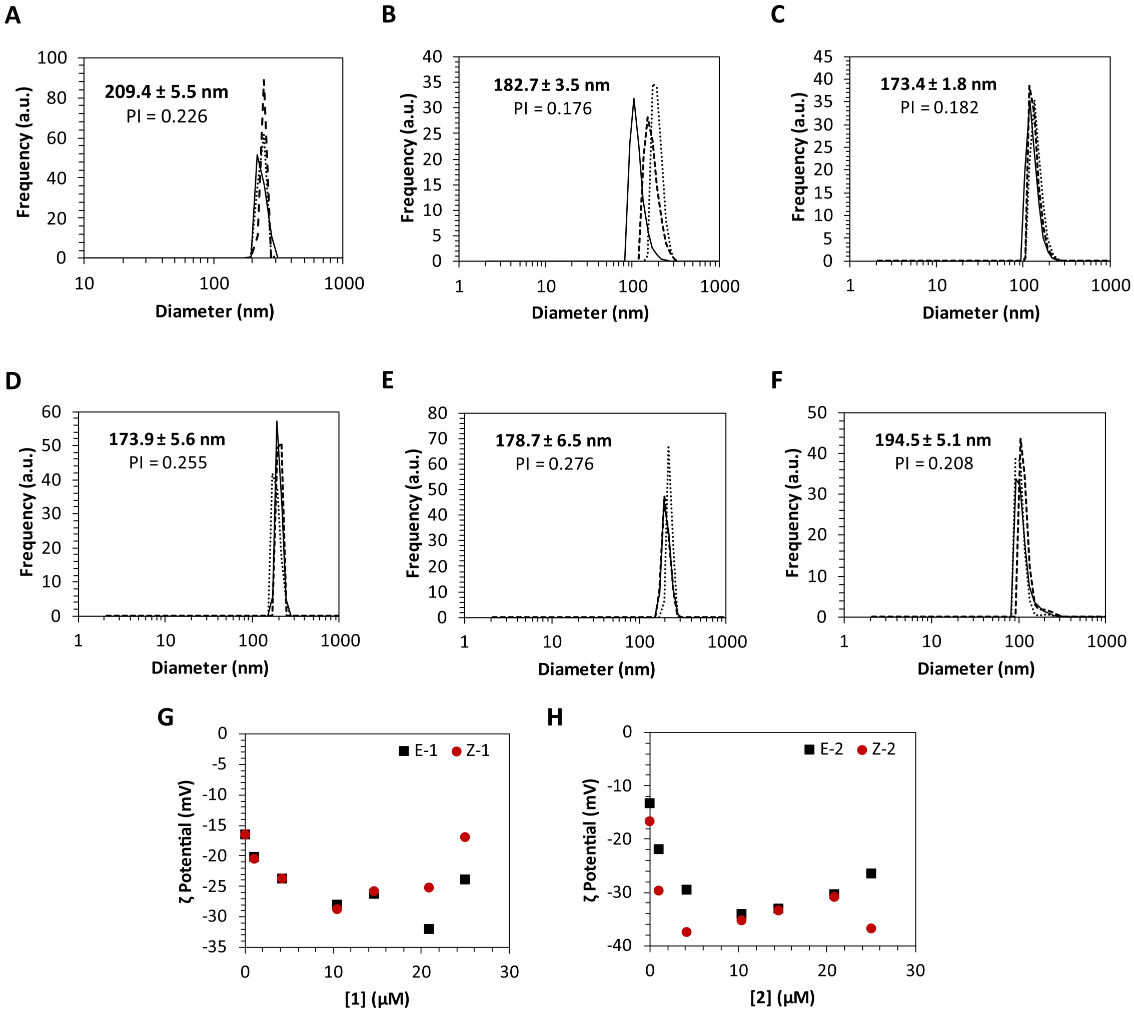


**Figure S34**- A) to F) correspond to the average size distribution of the EYPC-LUV with CF, synthesized for dye-efflux and light-activated release assays and respective Z-Average diameter: A) in the absence of peptide and counterion activator, in the presence of B) $R_6$, C) *E*-**2**, D) *Z*-**2**, E) $R_6$ - *E*-**2** and F) $R_6$ - *Z*-**2**. These assays were done in the same conditions as in the following fluorescence assays, with 15 μM of EYPC-LUV $\supset$ CF, 200 nM of counterion activator **2** and 4 μM of peptide. ζ-potential of the EYPC-LUV, at 260 μM, in the presence of increasing concentrations of both *E* and *Z* isomers of G) **1** and H) **2**. The decrease in ζ-potential indicates that upon addition of **1** and **2** to a LUV mixture, these incorporate into the liposome membrane.

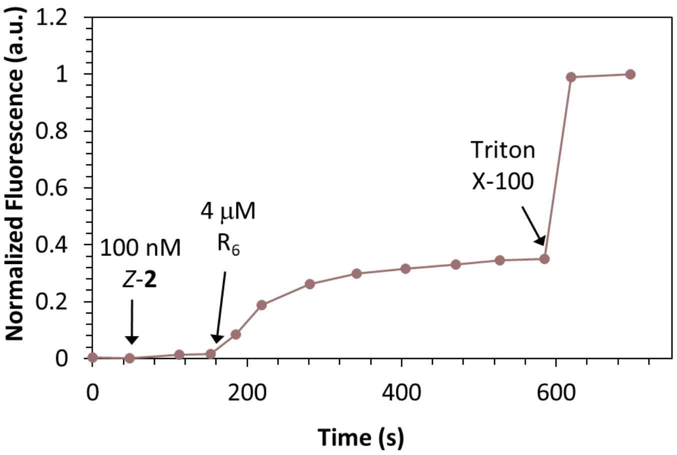


**Figure S35** - Example of release assay kinetics with addition of each element marked in the graph. The EYPC-LUV Ↄ CF concentration was maintained at 15 μM throughout these assays, with 50 mM concentration of CF inside the liposomes. The addition of the peptide initiates the transport activation and in the end of the assay, the measurement is normalized by the addition of Triton X-100, which destroys the vesicles and leads to the maximum of CF emission.

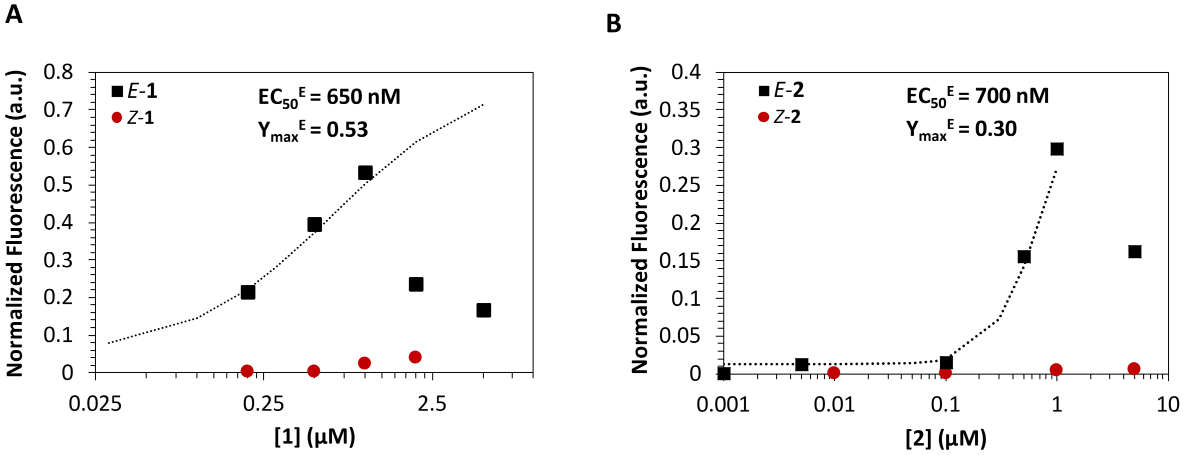


**Figure S36** - Dose-response curves of the activation of release of Carboxyfluorescein by the two calix-azo activators, A) **1** and B) **2**, in the presence of the peptide $R_4$ (5 μM). The EYPC-LUV Ↄ CF concentration was maintained at 15 μM throughout these assays, with 50 mM concentration of CF inside the liposomes. The efficient concentration at 50% release ($EC_{50}$) and maximum release of each of the activators' isomers (*E* and *Z* represented by black squares and red circles, respectively) is presented, as well as the respective fitted curves for the trans isomer; no considerable activation was observed for the cis isomers of both **1** and **2**.

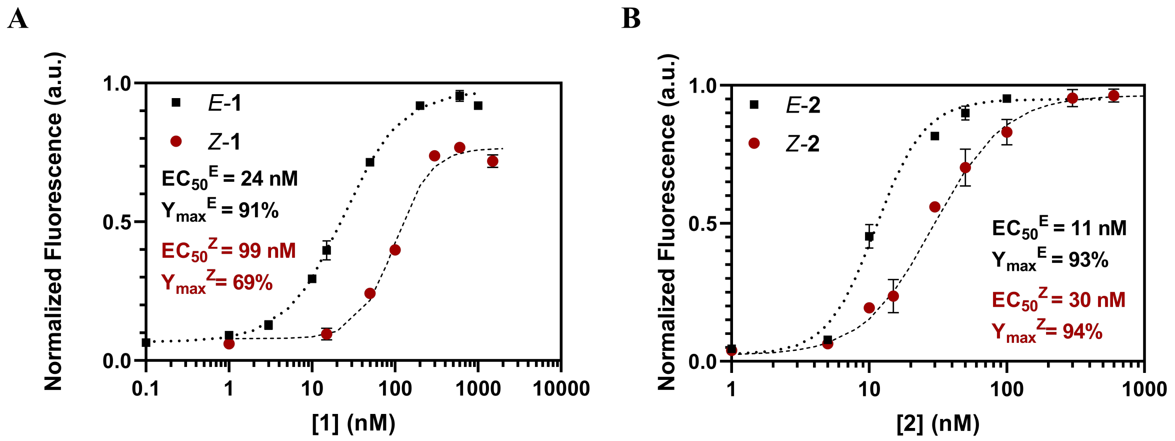


**Figure S37** - Dose-response curves of the activation of release of Carboxyfluorescein by the two calix-azo activators, A) **1** and B) **2**, in the presence of the peptide $R_8$ (4 µM). The EYPC-LUV ⊃ CF concentration was maintained at 15 µM throughout these assays, with 50 mM concentration of CF inside the liposomes. The efficient concentration at 50% release ($EC_{50}$) and maximum release of each of the activators' isomers (*E* and *Z* represented by black squares and red circles, respectively) is presented, as well as the respective fitted curves.

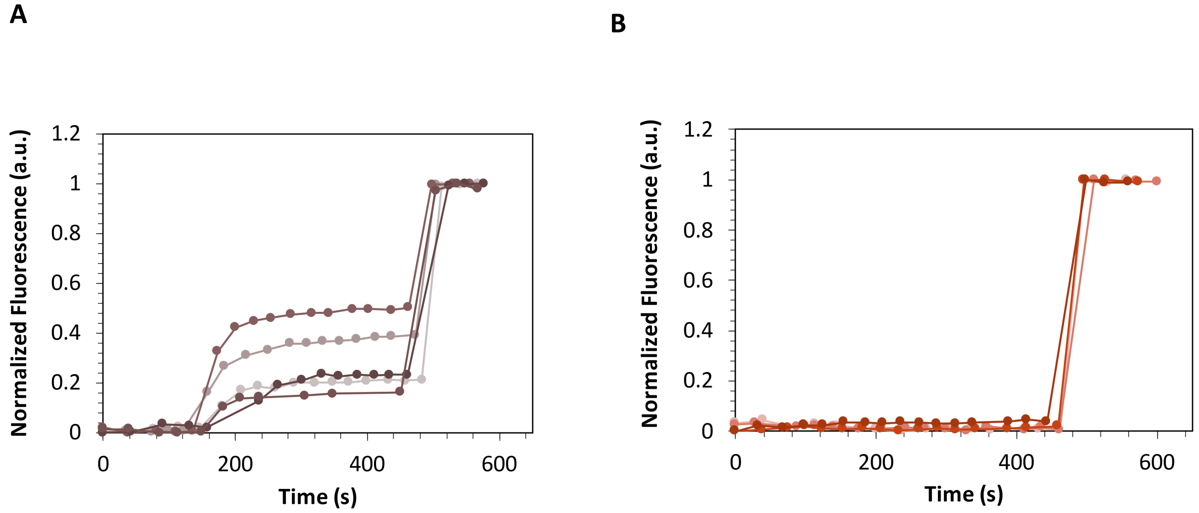


**Figure S38** - Release assay kinetics for each A) *E* and B) *Z*-**1** concentration represented in the dose response curves in Figure S36, in the presence of 5 µM of the peptide $R_4$. At approximately 80 s, the counterion activator is added, while the peptide is added at 140 s, which corresponds to the marked increase in emission, observed in A). For Z-**1**, no activation was observed in the presence of this peptide. The measurement is normalized with the addition of a small aliquot of Triton X-100 after stabilization of release of CF by the **1**-$R_4$ complex, to obtain the maximum emission of CF possible.

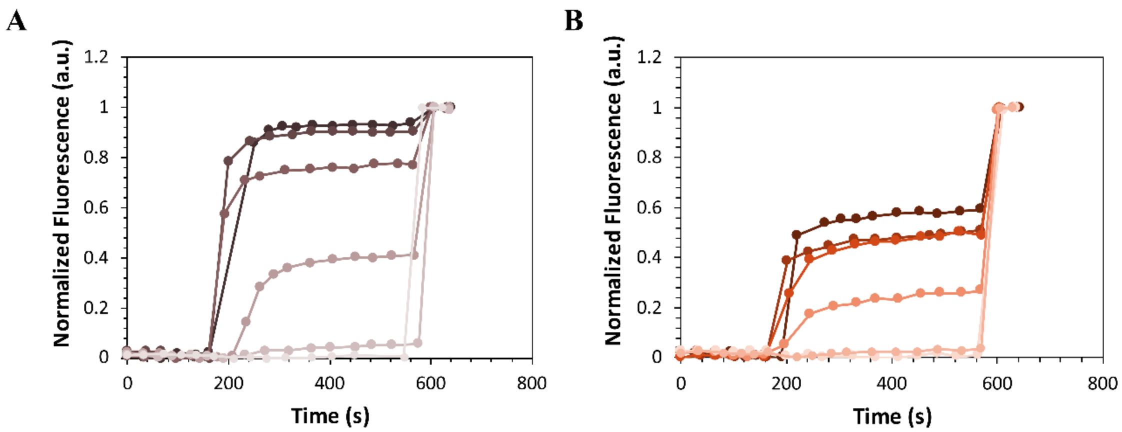


**Figure S39** - Release assay kinetics for each A) *E* and B) *Z*-**1** concentration represented in the dose response curves in Figure 3, in the presence of 4 µM of the peptide $R_6$. At approximately 80 s, the counterion activator is added, while the peptide is added at 140 s, which corresponds to the marked increase in emission, observed in both A) and B). The measurement is normalized with the addition of a small aliquot of Triton X-100 after stabilization of release of CF by the **1**-$R_6$ complex, to obtain the maximum emission of CF possible.

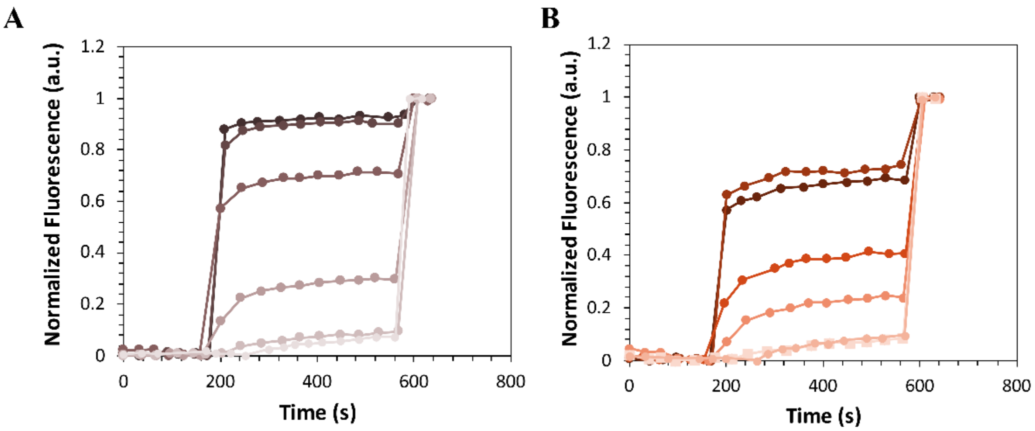


**Figure S40** - Release assay kinetics for each A) *E* and B) *Z*-**1** concentration represented in the dose response curves in Figure S37, in the presence of 4 µM of the peptide $R_8$. At approximately 80 s, the counterion activator is added, while the peptide is added at 140 s, which corresponds to the marked increase in emission, observed in both A) and B). The measurement is normalized with the addition of a small aliquot of Triton X-100 after stabilization of release of CF by the **1**-$R_8$ complex, to obtain the maximum emission of CF possible.

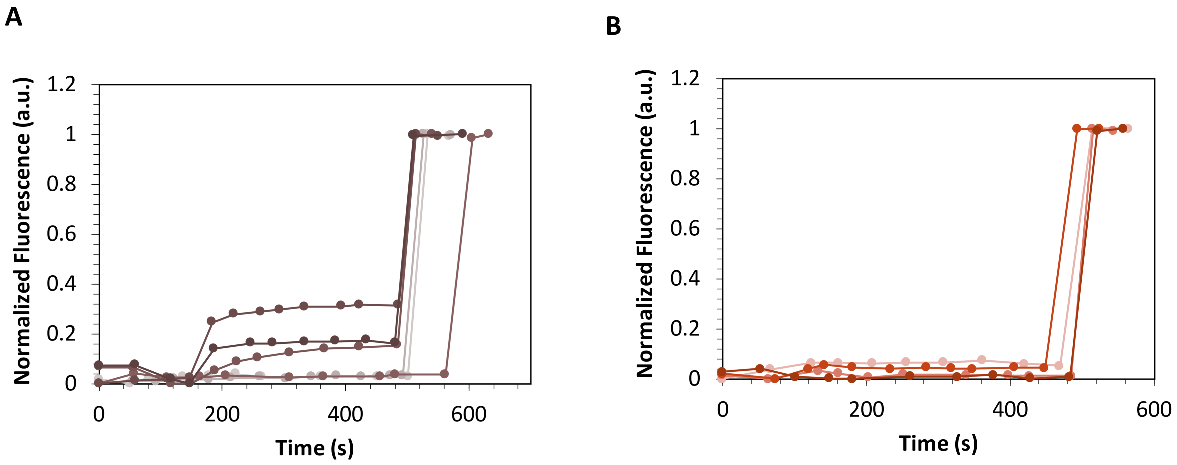


**Figure S41** - Release assay kinetics for each A) *E* and B) *Z*-**2** concentration represented in the dose response curves in Figure S36, in the presence of 5 µM of the peptide $R_4$. At approximately 80 s, the counterion activator is added, while the peptide is added at 140 s, which corresponds to the marked increase in emission, observed in A). For Z-**2**, no activation was observed in the presence of this peptide. The measurement is normalized with the addition of a small aliquot of Triton X-100 after stabilization of release of CF by the **2**-$R_4$ complex, to obtain the maximum emission of CF possible.

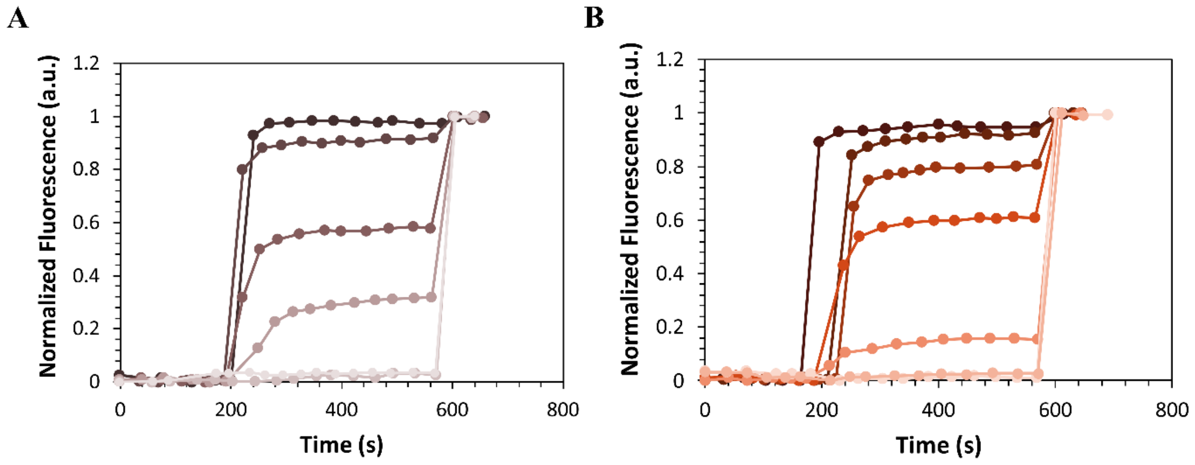


**Figure S42** - Release assay kinetics for each A) *E* and B) *Z*-**2** concentration represented in the dose response curves in Figure 3, in the presence of 4 µM of the peptide $R_6$. At approximately 80 s, the counterion activator is added, while the peptide is added at 140 s, which corresponds to the marked increase in emission, observed in both A) and B). The measurement is normalized with the addition of a small aliquot of Triton X-100 after stabilization of release of CF by the **2**-$R_6$ complex, to obtain the maximum emission of CF possible.

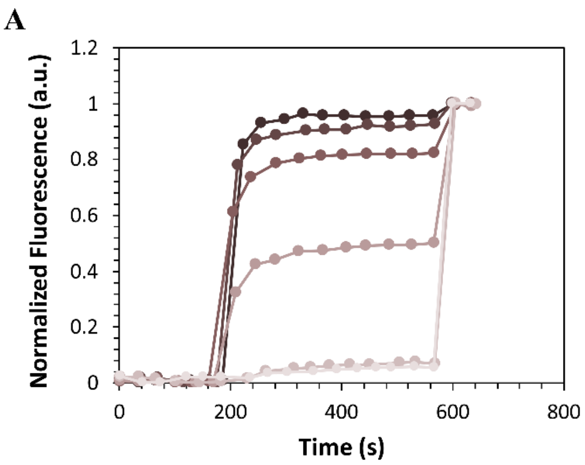


**Figure S43** - Release assay kinetics for each A) *E* and B) *Z*-**2** concentration represented in the dose response curves in Figure S37, in the presence of 4 µM of the peptide $R_8$. At approximately 80 s, the counterion activator is added, while the peptide is added at 140 s, which corresponds to the marked increase in emission, observed in both A) and B). The measurement is normalized with the addition of a small aliquot of Triton X-100 after stabilization of release of CF by the **2**-$R_8$ complex, to obtain the maximum emission of CF possible.

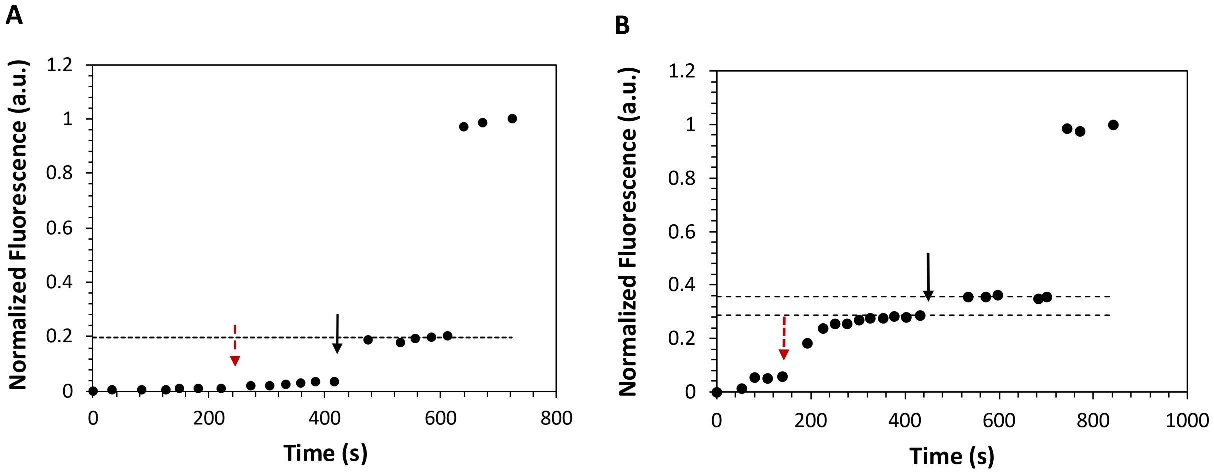


**Figure S44** - Light-modulated dye efflux assays done with the peptide A) $R_6$, 4 µM, and B) $R_8$, 5 µM, with a fixed concentration of *Z*-**1** of 0.20 µM for both assays. 40 µM of EYPC-LUV and 20 µM EYPC-LUV⊃CF (50 mM of CF) were used in the first assay and 30 µM of EYPC-LUV and 7 µM EYPC-LUV⊃CF (50 mM of CF) were used in the latter. The assay was performed in the same way as the dye release assays (addition of the peptide marked with a red dashed arrow) with an additional irradiation step for an interval of 30 to 40 min (marked with a black arrow), leading to an increase in release of ~15% for the peptide $R_6$ and only 7% for the peptide $R_8$.

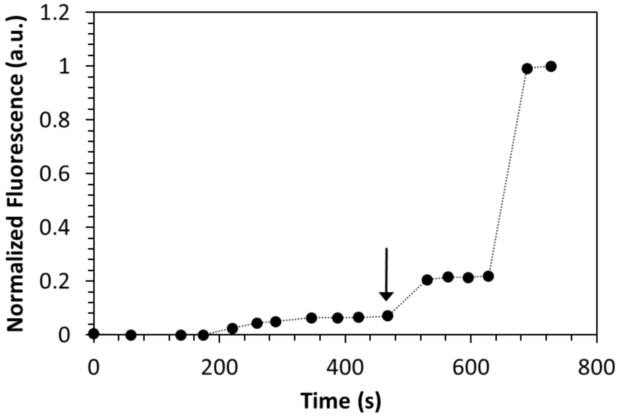


**Figure S45** - Light-modulated dye efflux assay done with the peptide $R_8$, 4 µM, with a fixed concentration of *Z*-**2** of 16 nM. 15 µM of EYPC-LUV⊃CF (50 mM of CF) were used and the light activation step was done with a single 25 min irradiation cycle at 500 nm (marked with a black arrow). The increase of release obtained was of 14%.

**A**

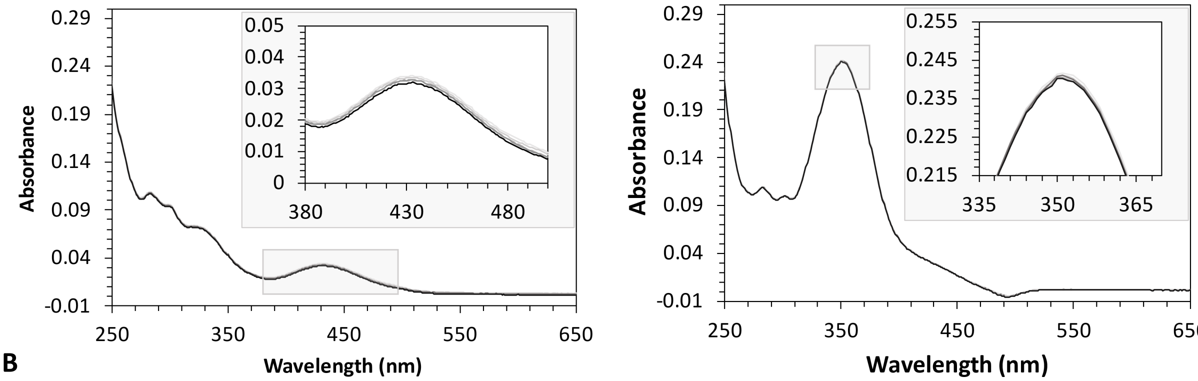


**B**

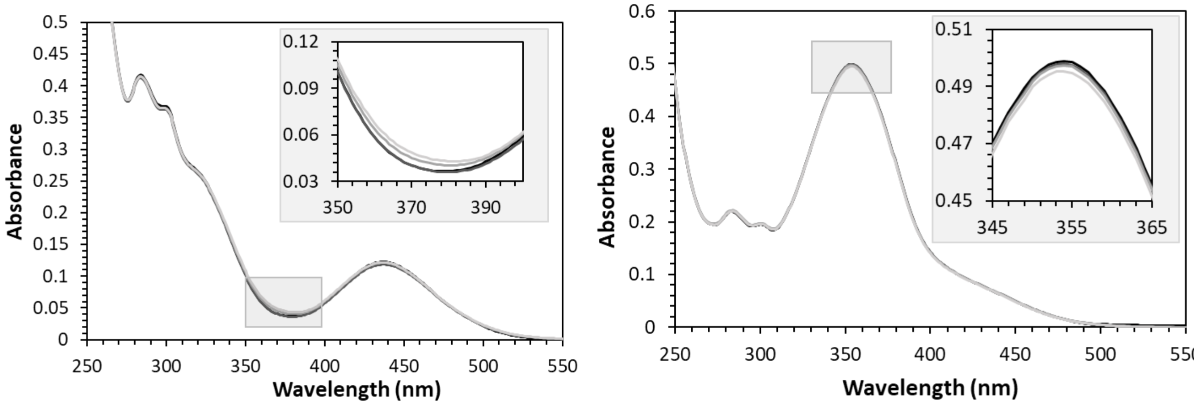


**Figure S46** - Absorption spectra of *Z* and *E*- A) **1** and B) **2** in the presence of EYPC-LUV⊃CF (lipid concentration at 12 µM and CF concentration of 50 mM inside the LUV), after irradiation in the fluorimeter, used for previous experiments – excitation slits fixed at 0.5 nm, window of measurement of 100 nm and integration time of 0.1 s. Black spectrum corresponds to the initial absorption spectrum and light grey to the spectrum after 25 spectra done in the fluorimeter in the acquisition settings described.

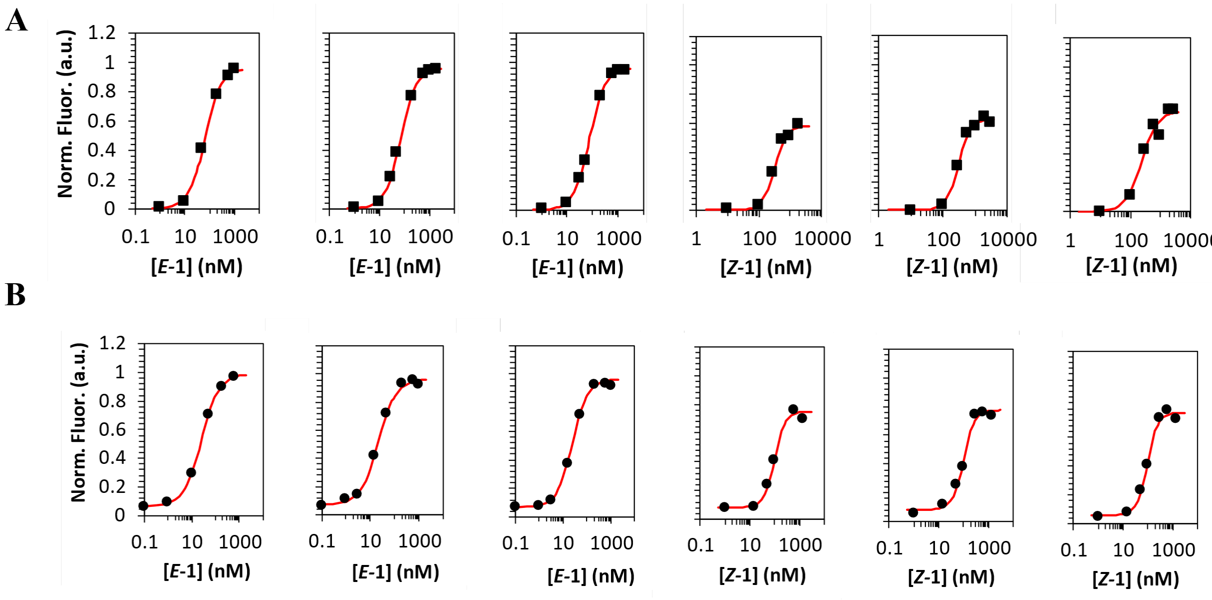


**Figure S47** - Triplicates of the dose-response curves for the peptides A) $R_6$ and B) $R_8$, with both isomers of the activator **1**. LUV concentration was fixed at 15 µM with 50 mM of CF encapsulated and peptide concentration was fixed at 4 µM.

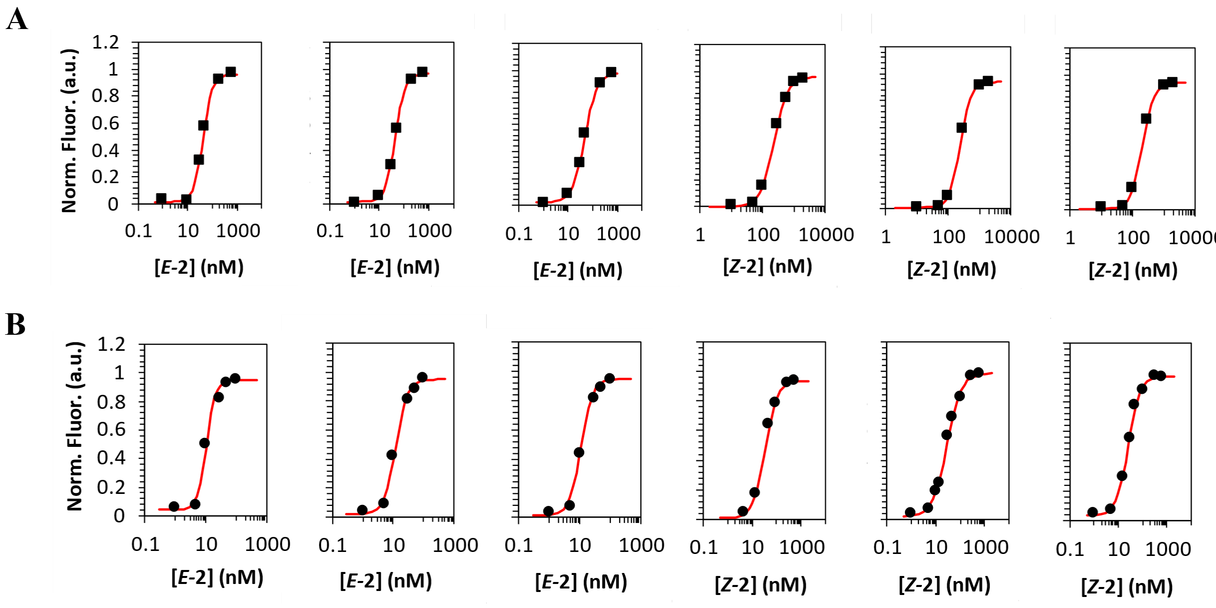


**Figure S48** - Triplicates of the dose-response curves for the peptides A) $R_6$ and B) $R_8$, with both isomers of the activator **2**. LUV concentration was fixed at 15 µM with 50 mM of CF encapsulated and peptide concentration was fixed at 4 µM.

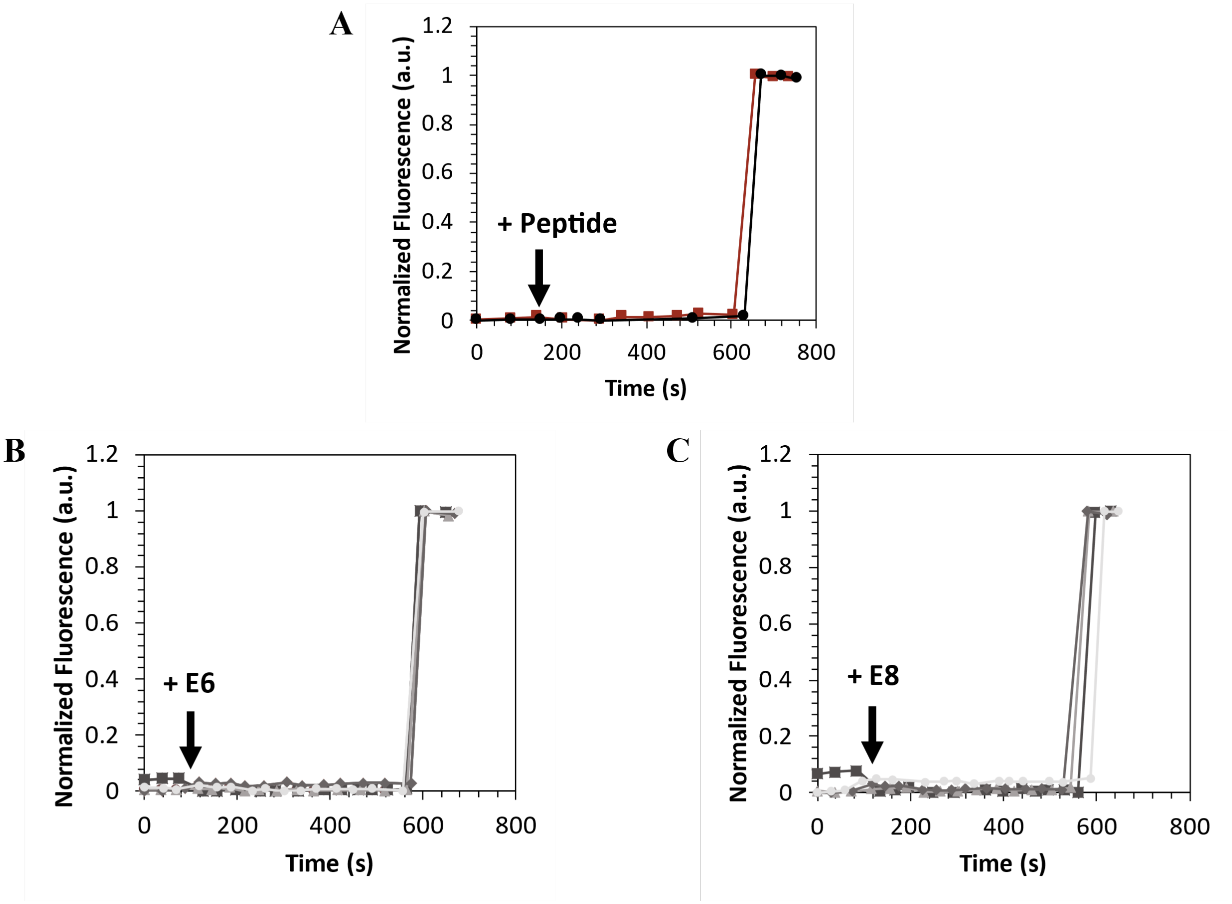


**Figure S49** – Dye release assay control experiments. A) Addition of both $R_6$ (black circles) and $R_8$ (red squares) at 4 µM to LUV solutions (15 µM of lipid, with 50 mM CF encapsulated) did not activate the transport of CF to the outside of the liposomes. B) and C) show the addition of anionic peptides that do not interact with the calixarene unit ($E_6$ and $E_8$, respectively) to a LUV (15 µM of lipid, with 50 mM CF encapsulated) and calixarene activator (*E*-**2** from 10 nM to 100 µM, from light to dark, respectively).

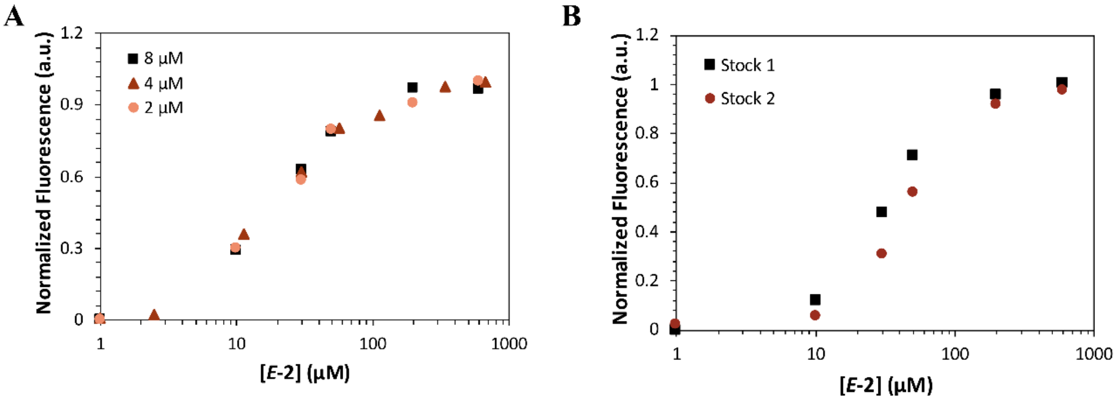


**Figure S50** – Dose response curves of the activation of release of CF by the calixarene activator **2** in the presence of A) different concentrations of peptide, $R_6$, and B) different LUV stocks at 15 µM lipid concentration in each stock.

## 6. Biological Assays.

**Cell lines and culture.** HeLa (cervix adenocarcinoma cells) cell line was obtained from ATCC and cultured in Dulbecco's Modified Eagle's Medium (DMEM; 4500 mg/L glucose, L-glutamine, sodium pyruvate, and sodium bicarbonate) supplemented with 10% FBS and 1% Penicillin-Streptomycin-Glutamine Mix (Fisher) (complete DMEM). They were maintained at 37 °C, 5% $CO_2$, and 95% humidity in an INCO 108 incubator (Memmert).

**Confocal microscopy assays.** HeLa cells were seeded the day before at 100,000 cells/mL (100 µL/well of a 96-well plate). Calixarenes *E*-**2** and *Z*-**2** (10, 50, 100, and 250 nM) and TAMRA-$R_8$ (3 µM) samples were diluted in DMEM stock and added to the cells (50 µL/well) for 25 min at 37 ºC, 5% $CO_2$. Before imaging, cells were treated with 1 µM Hoechst 33342 for 20 minutes to stain nuclei. Afterwards, cell culture media was removed, cells were washed with PBS, and 50 µL of complete DMEM without phenol red were added. Cells were immediately imaged using the 60× oil objective of a Dragonfly spinning disc confocal microscope mounted on a Nikon Eclipse Ti-E and equipped with an Andor Zyla 4.2 PLUS sCMOS digital camera. Fluorescence of Hoechst was excited with the 405 nm laser and the emission was detected at 450/50 nm; for the TAMRA fluorescence, an excitation of 561 nm was used while the emission was detected at 620/60 nm. Images were processed using ImageJ software (v1.52b).

**Flow cytometry assays.** HeLa cells were seeded the day before at 100,000 cells/mL (100 µL/well of a 96-well plate). Calixarenes *E*-**2** and *Z*-**2** (10, 50, 100, and 250 nM) and TAMRA-$R_8$ (3 µM) samples were diluted in DMEM stock and added to the cells (50 µL/well) for 25 min at 37 ºC, 5% $CO_2$. Immediately after incubation, cells were detached by replacing samples with 100 µL of Trypsin-EDTA for 10 min at 37 °C, 5% $CO_2$. Once cells were in suspension, trypsin was neutralized by the addition of 100 µL PBS containing 2% FBS and 5 mM EDTA. TAMRA fluorescence was excited at 532 nm (green laser) and

measured on a Guava easyCyte BG HT collecting the emission at 583/26 nm (Yellow-G channel), using InCyte v3.2 (GuavaSoft, Millipore). Cells with typical FSC and SSC parameters were selected and the mean fluorescence intensity was calculated for each sample. Each condition was measured in quadruplicate. Data analysis was performed with the InCyte software included in GuavaSoft 3.2 (Millipore).

**Cell viability assay.** HeLa cells were seeded the day before at 100,000 cells/mL (100 µL/well of a 96-well plate). Cells were then incubated with calixarene *E*-**2** or *Z*-**2** samples diluted in DMEM stock (0.01, 0.05, 0.1, 0.15, 0.25, 0.5, 0.75, 1, 2.5, 5, 7.5, 10, 15, 20, 40, 60, 80 and 100 µM, 50µL/well) for 24 hours. Afterwards, samples were removed and replaced with 100 µL of fresh complete DMEM supplemented with 0.5 mg/mL MTT. Cells were incubated for 2 hours before carefully removing the supernatant and dissolving the formazan crystals with DMSO (100 µL/well). The absorbance at 570 nm was measured with a plate reader (Tecan Infinite F200Pro) and the data normalized to the value of untreated cells (100% viability), after blank subtraction (cells previously treated with Triton X-100). Each condition was measured in triplicate. Curve fitting was performed with GraphPad Prism 6 software (v6.01) using a 4-parameters logistic model.

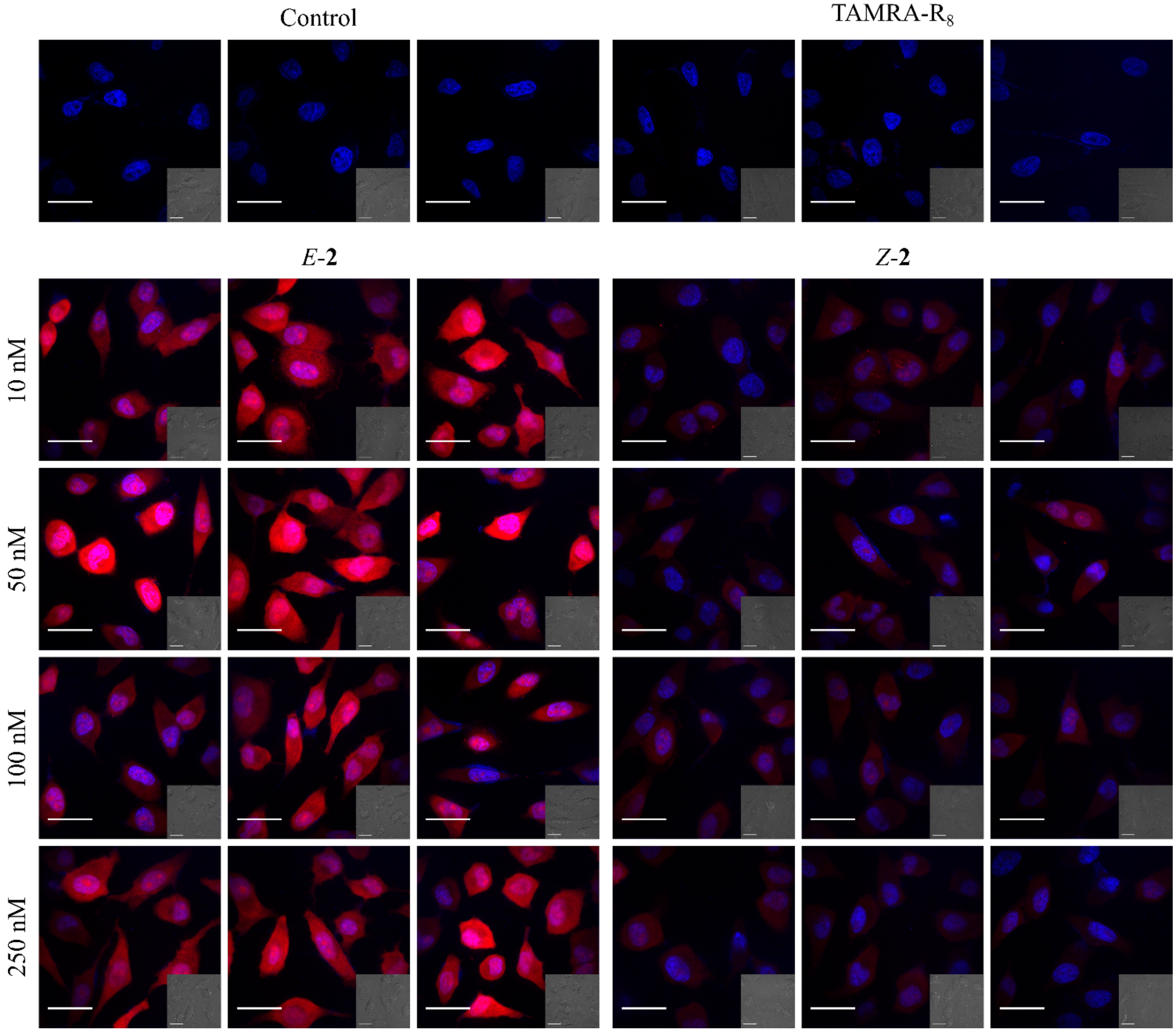


**Figure S51** - Confocal microscopy images of TAMRA-$R_8$ (in red) cellular uptake enhanced by *E*-**2** and *Z*-**2**. HeLa cells were incubated with TAMRA-$R_8$ (3 μM) in the absence (top row, right panel) or presence of several *E*-**2** and *Z*-**2** concentrations (10, 50, 100 and 250 nM) in DMEM stock for 25 min. Afterwards, nuclei were counterstained with Hoechst (in blue). Hoechst has been adjusted to similar intensities. Differential interference contrast (DIC) images in insets. Three representative images are presented for each condition. Scale bars, 50 μm.

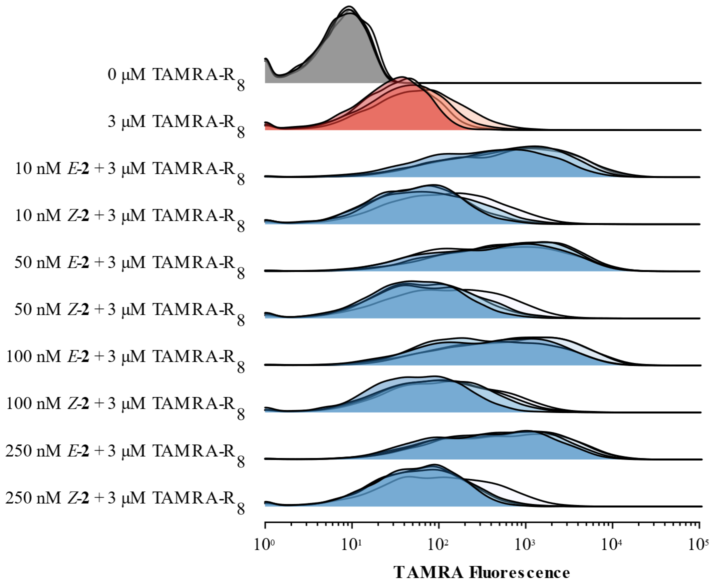


| | | R1 | R2 | R3 | R4 |
|---|---|---|---|---|---|
| | **Control** | 9.10 | 8.51 | 8.23 | 8.19 |
| | **TAMRA-R8** | 48.16 | 101.30 | 82.67 | 39.25 |
| ***E*-2** | **10 nM** | 1509.30 | 1625.74 | 1279.15 | 856.91 |
| | **50 nM** | 1144.89 | 1480.93 | 1347.63 | 1117.65 |
| | **100 nM** | 1569.76 | 1510.04 | 934.77 | 960.32 |
| | **250 nM** | 1249.76 | 1252.16 | 1041.01 | 849.96 |
| ***Z*-2** | **10 nM** | 119.15 | 160.44 | 94.49 | 83.65 |
| | **50 nM** | 137.94 | 155.80 | 99.42 | 80.02 |
| | **100 nM** | 229.26 | 181.69 | 107.07 | 73.67 |
| | **250 nM** | 108.17 | 120.96 | 89.64 | 79.71 |

**Figure S52** - Flow cytometry results of TAMRA-$R_8$ cellular uptake enhanced by *E*-**2** and *Z*-**2**. HeLa cells were incubated with TAMRA-$R_8$ (3 µM) in the absence or presence of several *E*-**2** and *Z*-**2** concentrations (10, 50, 100 and 250 nM) in DMEM stock for 25 min. Afterwards, cells were trypsinized and analyzed by flow cytometry. Left, normalized histograms showing TAMRA fluorescence from four replicates (curves in different colour tones) per sample. Right, TAMRA mean fluorescence intensity values for each sample and replicate (R1-R4).

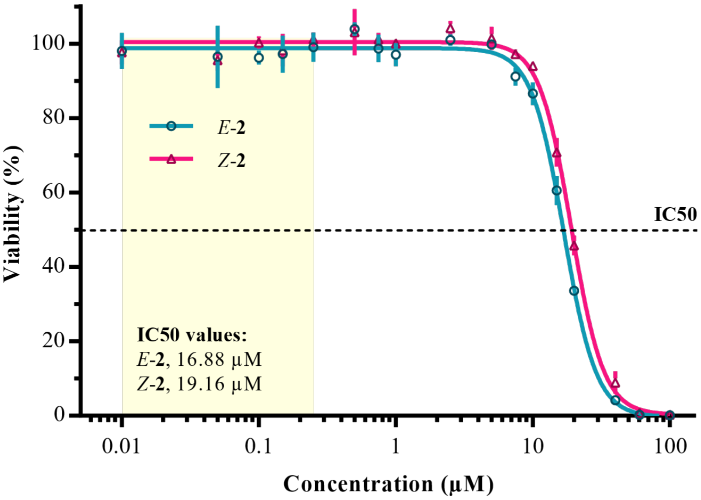


| $y = y_0+(y_{max}-y_0)/(1+(x/IC50)^n)$ | | | | |
|---|---|---|---|---|
| | *E*-2 | | *Z*-2 | |
| | Value | Error | Value | Error |
| $y_0$ | -0.364 | 1.179 | 0.274 | 1.342 |
| $y_{max}$ | 98.780 | 0.630 | 100.400 | 0.659 |
| IC50 | 16.880 | 0.324 | 19.160 | 0.428 |
| n | 3.650 | 0.259 | 3.691 | 0.306 |
| $R^2$ | 0.998 | NA | 0.998 | NA |

**Figure S53** - MTT viability assay at increasing concentrations of *E*-**2** and *Z*-**2**. HeLa cells were incubated with the samples in DMEM stock for 24 hours before the MTT addition. Curve fitting was performed with GraphPad Prism 6 software (v6.01) using a 4-parameters logistic model (right table). In yellow, region of concentrations employed in the delivery assays. $IC50_{E\text{-}2}$ = 16.88 µM; $IC50_{Z\text{-}2}$ = 19.16 µM.

# 7. References


(1) Montalti, M.; Credi, A.; Prodi, L.; Gandolfi, M. T. Chemical Actinometry. In *Handbook of Photochemistry*; Taylor & Francis Group, LLC, 2006; pp 601–617.

(2) Roibu, A.; Fransen, S.; Leblebici, M. E.; Meir, G.; Van Gerven, T.; Kuhn, S. An Accessible Visible-Light Actinometer for the Determination of Photon Flux and Optical Pathlength in Flow Photo Microreactors. *Sci. Rep.* **2018**, *8* (1), 1–10. https://doi.org/10.1038/s41598-018-23735-2.

(3) Basilio, N.; García-Río, L.; Martín-Pastor, M. Counterion Binding in Solutions of p -Sulfonatocalix[4]Arene. *J. Phys. Chem. B* **2010**, *114* (21), 7201–7206. https://doi.org/10.1021/jp101474a.

(4) Tkachenko, I. M.; Kobzar, Y. L.; Purikova, O. G.; Tolstov, A. L.; Shekera, O. V.; Shevchenko, V. V. Design, Synthesis and Photoisomerization Behavior of Novel Azobenzene-Based Dyes Containing Different Alkyl Chains and Isolation Groups. *Tetrahedron Lett.* **2016**, *57* (49), 5505–5510. https://doi.org/10.1016/j.tetlet.2016.10.101.

(5) Haiying, L.; Zhongfan, L. A Convenient Synthesis of Novel Mercapto-Ended Azobenzene Derivatives. *Synth. Commun.* **1998**, *28* (20), 3779–3785. https://doi.org/10.1080/00397919808004930.

(6) Merrifield, R. B. Solid Phase Peptide Synthesis. I. The Synthesis of a Tetrapeptide. *J. Am. Chem. Soc.* **1963**, *85* (14), 2149–2154. https://doi.org/10.1021/ja00897a025.

(7) Barba-Bon, A.; Pan, Y.; Biedermann, F.; Guo, D.; Nau, W. M.; Hennig, A. Fluorescence Monitoring of Peptide Transport Pathways into Large and Giant Vesicles by Supramolecular Host–Dye Reporter Pairs. *J. Am. Chem. Soc.* **2019**, *141* (51), 20137–20145. https://doi.org/10.1021/jacs.9b09563.

(8) Pazo, M.; Juanes, M.; Lostalé-Seijo, I.; Montenegro, J. Oligoalanine helical callipers for cell penetration. *Chem. Commun.* **2018**, *54* (50), 6919–6922. https://doi.org/10.1039/C8CC02304B
